# Synthesis and in silico inhibitory action studies of azo-anchored imidazo[4,5-*b*]indole scaffolds against the COVID-19 main protease (M^pro^)

**DOI:** 10.1038/s41598-024-57795-4

**Published:** 2024-05-06

**Authors:** Deepika Geedkar, Ashok Kumar, Pratibha Sharma

**Affiliations:** School of Chemical Sciences, Devi Ahilya University, Indore, Madhya Pradesh India

**Keywords:** Drug development, Medicinal chemistry, Chemistry, Medicinal chemistry, Organic chemistry, Theoretical chemistry

## Abstract

The present work elicits a novel approach to combating COVID-19 by synthesizing a series of azo-anchored 3,4-dihydroimidazo[4,5-*b*]indole derivatives. The envisaged methodology involves the l-proline-catalyzed condensation of *para*-amino-functionalized azo benzene, indoline-2,3-dione, and ammonium acetate precursors with pertinent aryl aldehyde derivatives under ultrasonic conditions. The structures of synthesized compounds were corroborated through FT-IR, ^1^H NMR, ^13^C NMR, and mass analysis data. Molecular docking studies assessed the inhibitory potential of these compounds against the main protease (M^pro^) of SARS-CoV-2. Remarkably, in silico investigations revealed significant inhibitory action surpassing standard drugs such as Remdesivir, Paxlovid, Molnupiravir, Chloroquine, Hydroxychloroquine (HCQ), and (N3), an irreversible Michael acceptor inhibitor. Furthermore, the highly active compound was also screened for cytotoxicity activity against HEK-293 cells and exhibited minimal toxicity across a range of concentrations, affirming its favorable safety profile and potential suitability. The pharmacokinetic properties (ADME) of the synthesized compounds have also been deliberated. This study paves the way for in vitro and in vivo testing of these scaffolds in the ongoing battle against SARS-CoV-2.

## Introduction

Since the beginning of 2020, the world has been navigating through a challenging situation with the emergence of ‘Severe Acute Respiratory Syndrome Coronavirus-2 (SARS-CoV-2) induced Novel Coronavirus Disease (COVID-19). This has infected more than 774.47 million people across 228 countries and territories, resulting in over 7.03 million deaths^1^. The phylogenetic analysis revealed that the causative pathogen of COVID-19 belongs to the subgenus sarbecovirus within the genus Betacoronavirus (βCoV)^2^. The SARS-CoV-2 is the seventh human pathogenic coronavirus among the other six related species namely 229E, NL63, OC43, HKU1, MERS-CoV (Middle East Respiratory Syndrome Coronavirus), and SARS-CoV^3^. The literature review reveals that the cross-species and human-to-human transmission of the virus is regulated by spike protein receptor-binding domain (RBD) and its host receptor (ACE2) similar to SARS-CoV caused a viral outbreak in 2002^4–8^.

A variety of therapeutic agents are available for the treatment of coronavirus infection and various existing FDA-approved anti-malarial and anti-viral drugs have been formulated as supportive care for the treatment of this pandemic infection^5,6^. Furthermore, apart from the existing FDA-approved drugs, several traditional medicines have also gained significant interest in the treatment of coronavirus infection across the globe. Therefore, a prompt development of novel compounds as a potential therapeutic agent against COVID-19 caused by SARS-CoV-2 is a significant pursuit. The development and discovery of potential therapeutic agents for a specific disease through traditional methods is expensive and time-consuming. However, computer-aided in silico techniques such as molecular docking and molecular dynamics are around to be better alternatives for the designing and development of new potential inhibitors for specific diseases.

**Figure 1:**
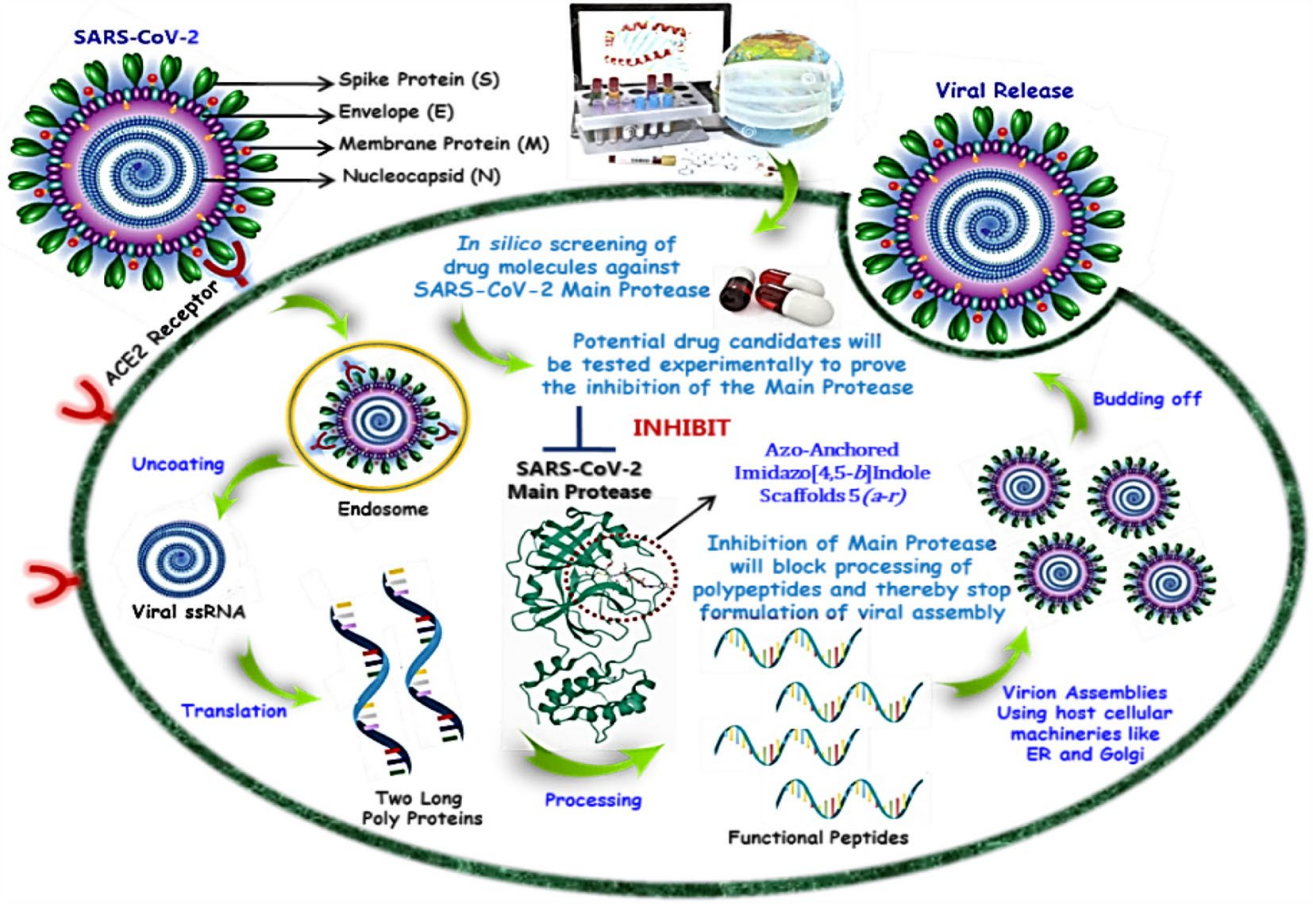
Proposed mechanism of inhibition of viral replication by azo-anchored 3,4-dihydroimidazo[4,5-*b*] indole derivatives **5*(a–r)*** against SARS-CoV-2 main protease (M^pro^).

In addition to finding the vaccines against COVID-19, there are several ongoing searches for therapeutics acting on SARS-CoV-2^9–12^. Depending on the activity, the therapies against SARS-CoV-2 can be classified into two categories; one is acting on the human immune system, and the other acts on the coronavirus itself. The latter can be further sub-classified into two categories; one prevents the viral RNA synthesis and replication and the second blocks the binding to human cell receptors. The life cycle of the coronavirus begins with the binding of the S protein of the virus to its receptor on the host cells, the ACE2 (Angiotensin-Converting Enzyme-2). The binding is then followed by the fusion of the viral envelope with the host cell membrane and the release of the viral genome into the cytoplasm. The viral genome (+ ssRNA) confines the host ribosomes to translate into 16 non-structural proteins (NSPs) upon auto proteolytic cleavage by two proteases such papin-like proteases (PL^pro^) and 3-chymotrypsin like protease (3CL^pro^). The structural proteins (S, E, M, and N) are translated by a sub-genome (Sg) translation mechanism. The sub-genome (Sg) is produced through discontinuous transcription of viral RNA. After the effective completion of genome replication and translation, mature proteins congregate alongside the positive-sense single-stranded RNA genome, culminating in the creation of a novel virion^13–16^. The proposed inhibition mechanism of viral replication by azo-anchored 3,4-dihydroimidazo[4,5-*b*] indole derivatives **5*(a–r)*** against SARS-CoV-2 is depicted in Fig. 1.

The 3-chymotrypsin-like protease (3CL^pro^) also recognized as the main protease (M^pro^) of novel coronavirus (SARS-CoV-2) plays a significant role in viral replication in the life cycle of coronavirus^6,14,17–19^. Thus, its inhibition could afford a promising therapeutic benchmark for developing an effective and specific treatment against COVID-19 infection. Many computational approaches have been explored for the analogous screening of potential inhibitors against SARS-CoV-2 main protease (M^pro^)^9,10^. Some protease inhibitors, for instance, Remdesivir, Paxlovid, Molnupiravir, Favipiravir, Chloroquine, and Hydroxychloroquine have been formally approved as potent drugs against SARS-CoV-2^20–22^.

**Figure 2:**
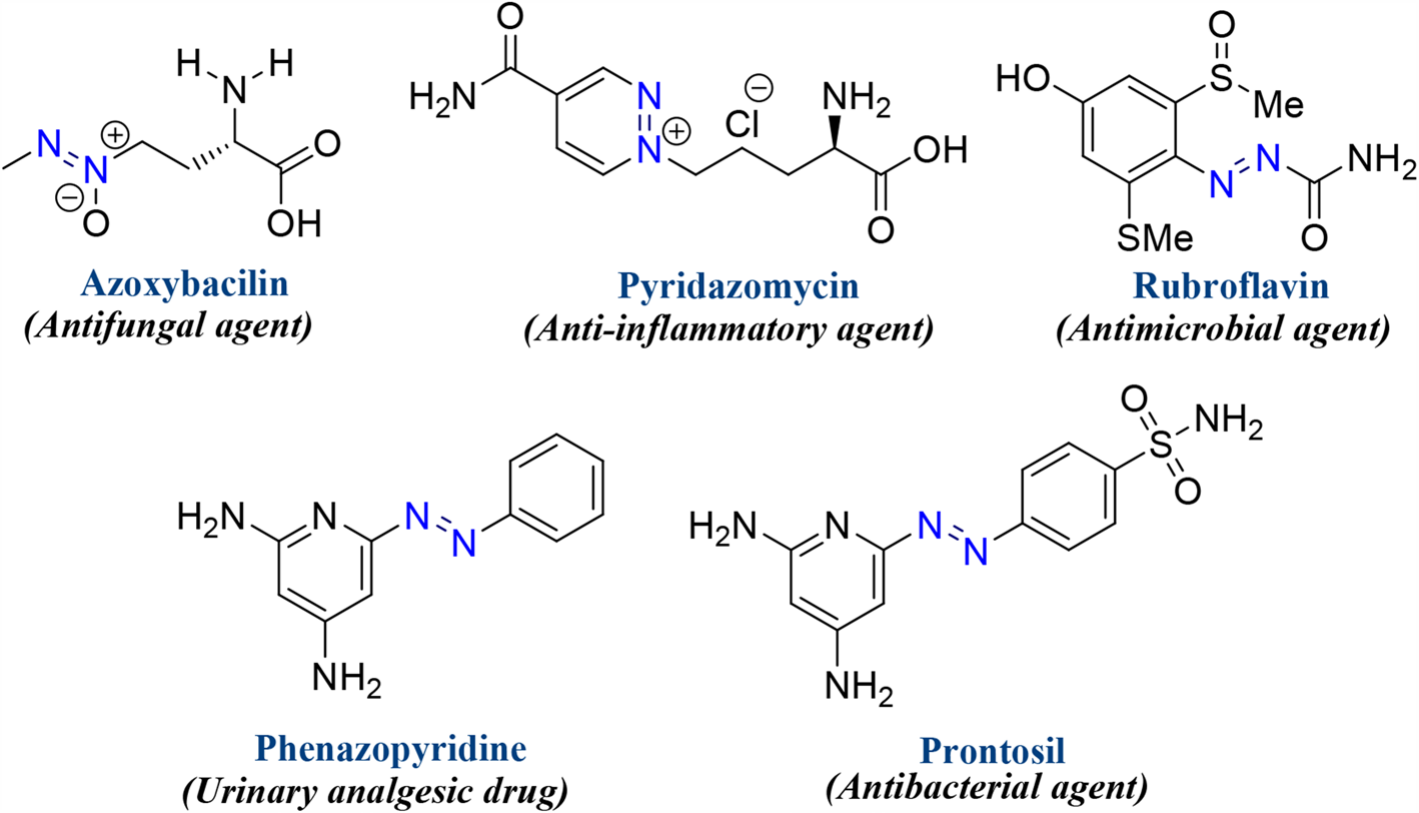
Some representative examples of biologically active azo-containing compounds and FDA-approved drugs.

Intriguingly, the nitrogen-embracing heterocyclic framework is the most prevalent structural motifs found in pharmaceutical chemistry as biologically active synthetic equivalents and natural products^23–25^. Subsequently, the development of imidazole-based therapeutic agents has acquired significant attention on account of their synthetic and effective biological significance. Their chemistry has gained substantial interest in recent years owing to their contribution to various medicinal applications viz*.* as potent anti-viral, anti-convulsant, anti-depressant, anti-cancer, anti-bacterial, anti-hypertensive, and prominent anti-inflammatory agents^26–30^. Moreover, the derivatives comprising conjugated chromophoric azo (–N=N–) functionality have acquired unique significance on account of their versatile applicability ranging from the dyeing of textile fiber, the coloring of varied polymers and plastics, cosmetics industries, biological-medical studies, and advanced application in organic synthesis^31–34^. Besides, the natural diazo alkaloid compounds are found in many microorganisms, marine organisms, plant parts, fungi, and ascomycetes, etc.^35^. However, despite possessing diversified biological activities, they have been utilized as anti-viral, anti-bacterial, anti-fungal, anti-tumor, anti-hypertensive, and anti-inflammatory therapeutic agents^31^. The structures of some naturally occurring azo alkaloid compounds and FDA-approved drugs are delineated in Fig. 2.

The rationale behind this research lies in the urgent necessity to address the global pandemic caused by SARS-CoV-2, marked by widespread infection and mortality rates, necessitating immediate development of effective treatments. Despite some relief provided by existing FDA-approved drugs and traditional medicines, the continuous evolution of the virus underscores the importance of exploring novel therapeutic avenues. Conventional approaches to drug development are time-consuming and costly, prompting the search for expedited approaches to identify potential treatments. Leveraging computer-aided in silico techniques as molecular docking facilitates rapid screening of compounds targeting the main protease (M^pro^) of SARS-CoV-2, essential for viral replication. By inhibiting M^pro^, the viral life cycle can be disrupted, potentially impeding virus spread within hosts.

The design and synthesis of novel compounds, particularly those incorporating imidazole scaffolds with azo functionality, present promising avenues for developing M^pro^ inhibitors with diverse biological activities^36–41^. Moreover, employing ultrasonic-assisted synthesis aligns with sustainable practices in medicinal chemistry. Furthermore, the incorporation of azo moieties in the imidazole fused indole ring could result in the development of compounds possessing more diversified biological activities. Therefore, buoyed by these findings, it was deemed worthwhile to synthesize a series of compounds embracing a fused imidazole framework^42–45^. The protocol endorsed an ultrasonic-assisted synthesis of novel azo-anchored 3,4-dihydroimidazo[4,5-*b*]indole derivatives **5*(a–r)***. An in silico attempt has been made to provide insight into the inhibitory potential of these novel azo imidazole derivatives against the main protease (M^pro^) of SARS-CoV-2, further bolstering the research’s significance in identifying effective therapeutic agents against COVID-19 and contributing to pandemic management and preparedness for future viral threats.

## Experimental section

### General procedure for the synthesis of *para*-amino functionalized azo benzene (1)

The aniline ***(a)*** (0.01 mol) was dissolved in a mixture of 4.0 ml of concentrated hydrochloric acid (HCl) and 4.0 ml of distilled water. The amine hydrochloride solution ***(b)*** was shaken vigorously and kept at freezing temperature. To this, an aqueous solution of sodium nitrite (NaNO_2_) 0.69 g (0.01 mol) in 5.0 ml of distilled water was added dropwise with continuous stirring, keeping the temperature of the reaction vessel between 0 and 5 °C. Meanwhile, in another beaker, 6.8 g (0.05 mol) of sodium acetate in 13.0 ml of distilled water was taken and cooled in an ice bath. Now the diazotized solution was added to this solution dropwise with thorough stirring. The reaction mixture was kept overnight, filtered under suction, washed thoroughly with cold water, dried, and recrystallized from a mixture of dimethylformamide (DMF) and ethyl alcohol (EtOH) (3:7) to give the corresponding yield of yellow crude diazoaminobenzene ***(c)***^46^.

**Scheme 1:**
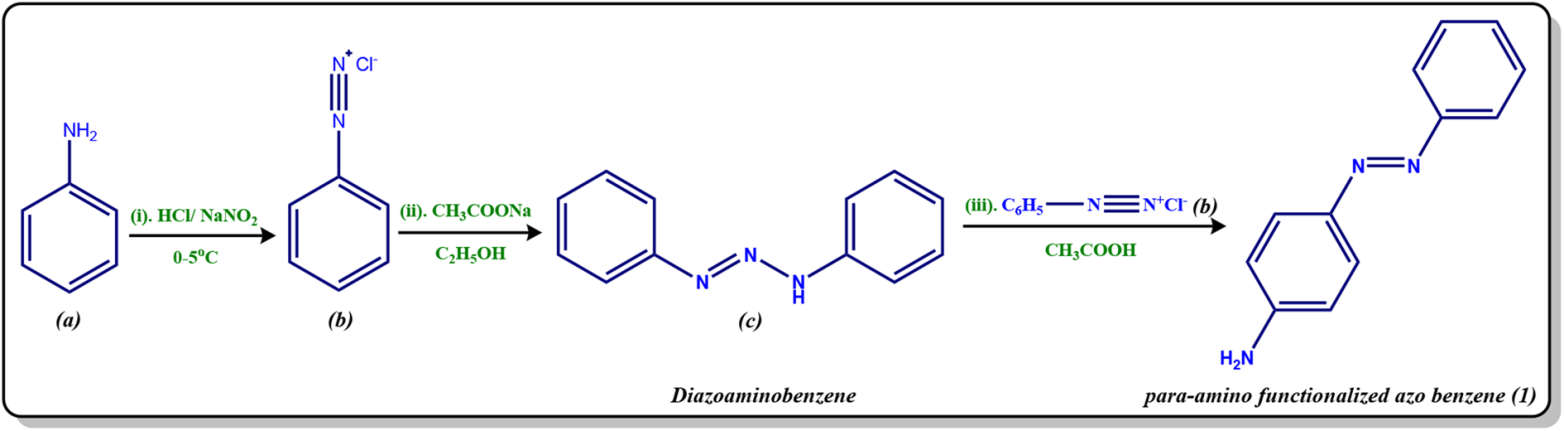
Synthesis of *para*-amino functionalized azo benzene ***(1).***

Now, the synthesized diazoaminobenzene ***(c)*** (1.0 g) was dissolved in amine hydrochloride solution ***(b)*** with constant stirring and kept at freezing temperature. Then, the glacial acetic acid (3.0 ml) with an equal volume of water was added with stirring, in order to remove the excess aniline in the form of its soluble acetate. The resulting mixture was kept overnight, followed by filtration, cold-water washing, drying, and subsequent recrystallization from carbon tetrachloride (CCl_4_) to yield the desired *para*-amino functionalized azo benzene ***(1)*** (Scheme 1).

#### The optimal reaction conditions

Step 1: Formation of amine hydrochloride solution ***(b)***: Aniline ***(a)*** (0.01 mol), Concentrated HCl (4.0 ml), Distilled water (4.0 ml); Step 2: Diazotization: Aqueous solution of NaNO_2_ (0.69 g, 0.01 mol) in distilled water (5.0 ml), at 0–5 °C; Step 3: Addition of diazotized solution to sodium acetate: CH_3_COONa (6.8 g, 0.05 mol) in water (13.0 ml), Step 4: Synthesis of *para*-amino functionalized azo benzene ***(1)***: Synthesized diazoaminobenzene ***(c)*** (1.0 g), Amine hydrochloride solution ***(b)***, Glacial acetic acid (3.0 ml) and Water (3.0 ml).

### General procedure for the synthesis of azo-anchored 3,4-dihydroimidazo[4,5-*b*]indole 5*(a–r)*

**Scheme 2:**
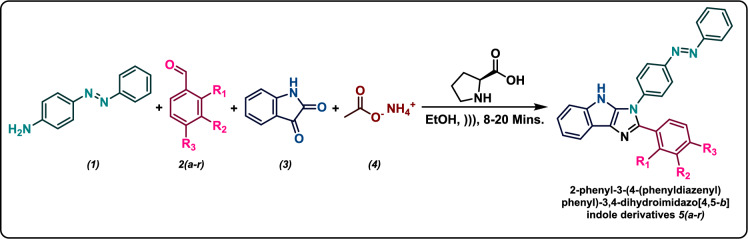
Ultrasonic-assisted synthesis of azo-anchored 3,4-dihydroimidazo[4,5-*b*]indole derivatives **5*(a–r)***.

The mixture of synthesized *para*-amino functionalized azo benzene **(*1*)** (1 mmol), pertinent aryl aldehyde derivatives ***2*(*a–r*)** (1 mmol), indoline-2,3-dione **(*3*)** (1 mmol), ammonium acetate** (*4*)** (1 mmol) and a catalytic amount of l-proline (5 mol%) in the presence of absolute ethanol (10 ml) was irradiated through an ultrasonic wave at room temperature for the ambient time (8–20 min)^6^. As the reaction time is very short, there was not a remarkable elevation of temperature due to ultrasonic shock. The ultrasonic apparatus utilized showed the temperature automatically so the temperature was controlled and fixed at room temperature by a water circulator in the case of any elevation of temperature^43^. The progress of the reaction was monitored through TLC (on aluminum sheets precoated with silica) using *n*-hexane/ethyl acetate (4:1) as the eluting system. After the completion of the reaction, the solvent was removed by rotary evaporation. The resultant mixture was then diluted with distilled water and subjected to extraction with ethyl acetate (3 × 25 ml). The organic layer was dried over anhydrous magnesium sulphate and concentrated under a vacuum. The crude product was washed with a little amount of *n*-hexane and recrystallized from ethanol solution to afford the products in good yields (Scheme 2). All the synthesized compounds are > 95% pure as ascertained by column chromatography/thin layer chromatography followed by elemental analyses. The analytical and spectroscopic data for each of the synthesized azo-anchored 3,4-dihydroimidazo[4,5-b]indole derivatives ***5(a–r)*** are summarized in Supporting Information.

#### Reaction condition

The mixture of 4-amino functionalized azo benzene (1 mmol) **(*1*),** pertinent aryl aldehyde derivatives (1 mmol) ***2*(*a–r*),** indoline-2,3-dione (1 mmol)** (*3*)**, ammonium acetate (1 mmol) **(*4*)** and a catalytic amount of l-proline (5 mol%) were sonicated in the presence of ethanol (10 ml).

### Preparation of protein and ligand for docking study

The X-ray crystallographic structures of the main protease (M^pro^) of SARS-CoV-2 (PDB ID: 6LU7) with a resolution of 2.16 Å have been retrieved from the Research Collaboratory for Structural Bioinformatics–Protein Data Bank (RCSB–PDB)^47^. The Molegro Virtual Docker (MVD 2013.6.0.0 evaluation version)^48^, was used for performing docking studies, which are based on molecular docking (MD) simulations viz. ligand and macromolecular interaction energy. The docking score function (E_score_), is defined by the following energy terms,

$${E}_{score}={E}_{inter}+{E}_{intra},$$

where *E*_*inter*_ is the ligand–protein interaction energy, *E*_*intra*_ is the internal energy of the ligand

$${E}_{inter}={\sum }_{i\in ligand}\cdot {\sum }_{j\in protein}\cdot \left({E}_{PLP}\left({r}_{\mathit{ij}}\right)\cdot +332.0\cdot \frac{{q}_{i}{q}_{j}}{4{{r}^{2}}_{ij}}\right).$$

The summation considers all the heavy atoms of the ligand and protein, wherein the cofactor atoms and water molecules have also been taken into consideration, if present; whereas the electrostatic interactions between charged atoms are considered by the second term.

$${E}_{intra}={\sum }_{{\text{i}}\in {\text{ligand}}}\cdot {\sum }_{{\text{j}}\in {\text{protein}}}\cdot {{\text{E}}}_{{\text{PLP}}}\left({{\text{r}}}_{{\text{ij}}}\right)\cdot +{\sum }_{\text{flexible bonds}}\text{ A }\left[1-\text{cos }\left({\text{m}}\times\uptheta -{\uptheta }_{0}\right)+ {E}_{clash}\right].$$

**Figure 3:**
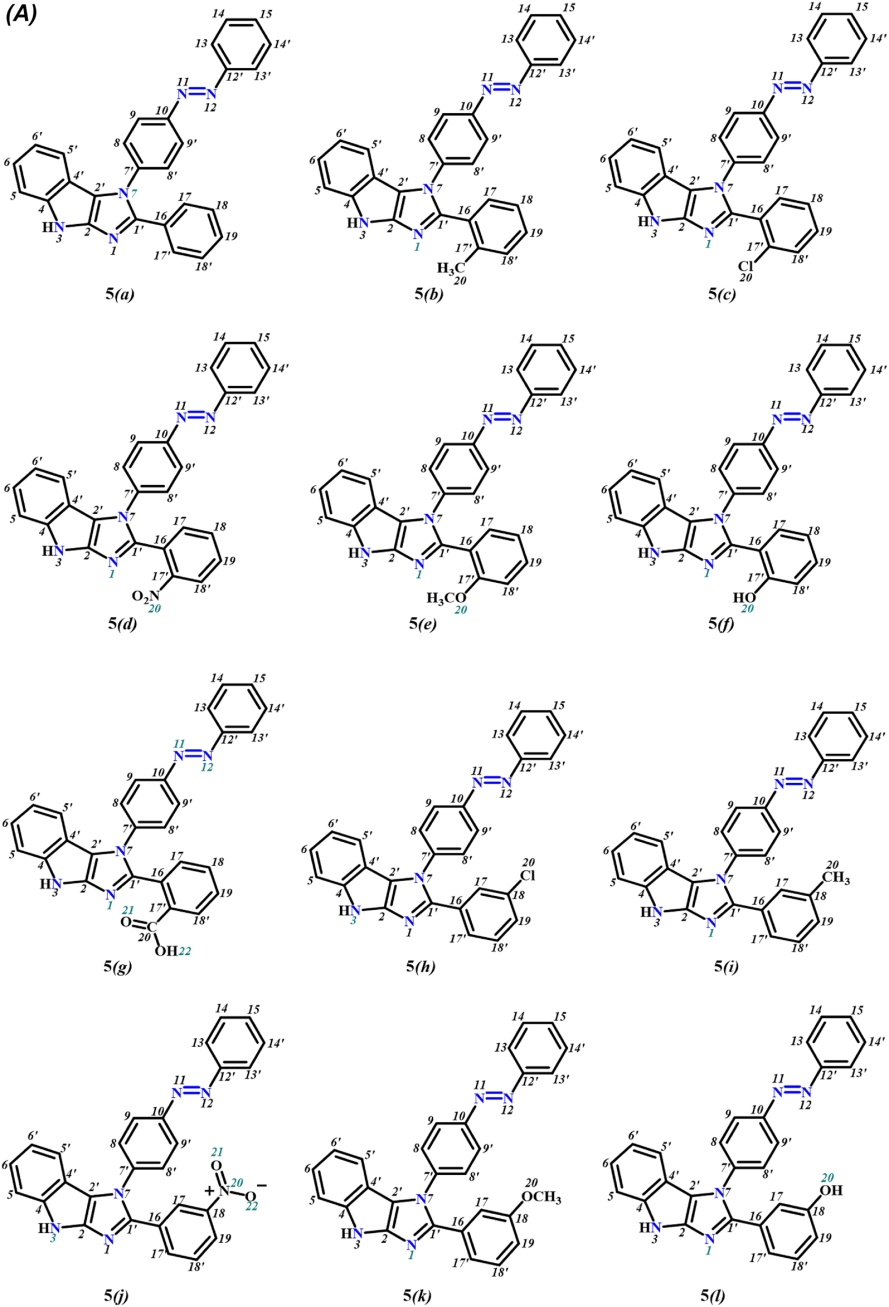

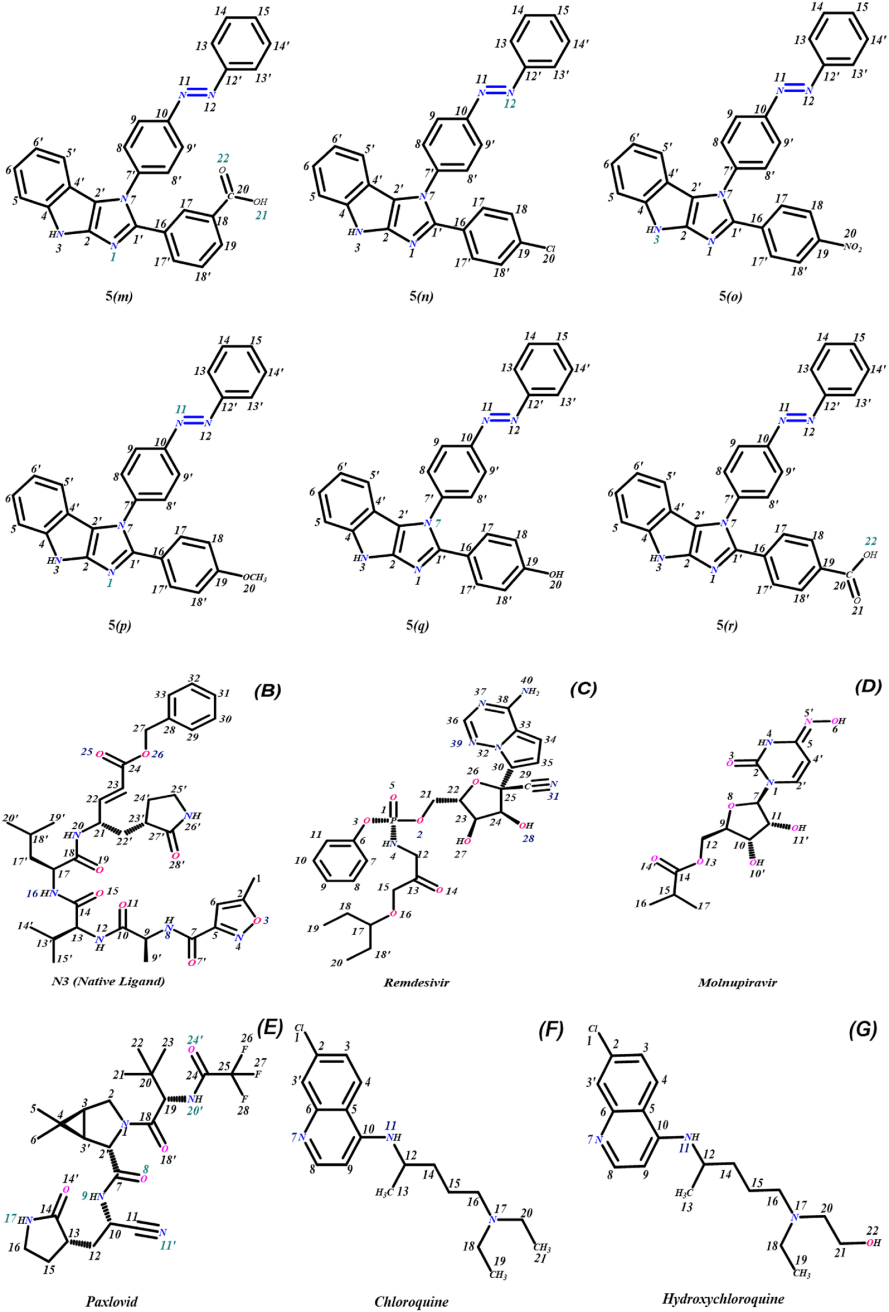
(**A**) Structure of synthesized ligands **5*(a–r)***, (**B**) inhibitor (N3), (**C**) Remdesivir, (**D**) Paxlovid, (**E**) Molnupiravir, (**F**) Chloroquine, and (**G**) Hydroxychloroquine.

Initially, the geometrically optimized three-dimensional structures of azo-anchored 3,4-dihydroimidazo[4,5-*b*]indole derivatives **5*(a–r)***, native ligand, and standard drugs were imported into the workspace of MVD along with the main protease protein, retrieved from the RCSB Protein Data Bank for performing MD simulations. The geometrical optimizations of compounds were attained by performing the molecular mechanics (MM2) and Hamiltonian approximation (AM1) optimizers until the root-mean-square (RMS) gradient value reaches a value smaller than 0.001 kcal mol^−1^ Å^−1^^49,50^. The chemical structure of azo-anchored 3,4-dihydroimidazo[4,5-*b*]indole derivatives **5*(a–r)***, inhibitor (N3) of the main protease protein and all the standard drugs are depicted in Fig. 3.

During the import process of the target, all the crystallographic water molecules and ions were removed from it. All the ligands and targets were processed with the protein preparation wizard present at the preparation window in the workspace of MVD, followed by the identification and detection of active sites (cavities) within the target protein. During this computational process, the maximum number of cavities was set to five, the grid resolution to 0.80 Å, and the probe size to 1.2 Å; while the other parameters were retained as default^49^. Consequently, the docking scores reveal the significant hydrogen bonding and interactions between the ligand of different conformations and key amino acid residues in the binding pocket of the target. Moreover, the MolDock score is the sum of internal ligand energies, protein interaction energies, and soft penalties. The protein–ligand energy is the total interaction energy between the ligand and the target molecule, whereas the steric score indicates the interaction energy between the ligand and protein. The H-Bond score is the hydrogen bonding energy between the protein and ligand. The re-ranking score function is computationally more valuable than the scoring function used during the docking simulations. In general, the re-rank score is better than the docking score function for determining the best pose among the various poses derived from the identical ligand^49,51^.

The active sites or cavities (1–5) with diverse surface areas and volumes have been depicted within the selected target for screening using the detect cavity module. The MD simulations were performed within the cavity of the larger surface area of protein. Some other parameters such as binding radius, grid resolution, and maximum iteration parameters were set to 15 Å, 0.3 Å, and 1500, respectively. The docking algorithm was set to MolDock Simplex Evolution (MolDock SE) docking algorithm with a population size of 50. For cluster similar poses and ignore similar poses (for multiple runs only), the RMSD thresholds were firm to 1.00 Å. While the selected number of independent runs was 10 and each of these runs was recurred to a single final solution (Pose). After the completion of docking simulations, only the negative lowest-energy representative cluster was recurred from each of them, followed by the removal of all the similar poses and keeping the best scoring pose. The clusters were ranked in order of increasing binding energy of the lowest binding energy conformation in each cluster. The analysis of the molecular docking results was carried out on the first binding free energy pose with minimum energy^49^.

### In vitro cytotoxicity assessment by MTT assay

The HEK-293 cells (human embryonic kidney cells) utilized in the present study were procured from the cell culture repository of NCCS, Pune, India. Cells were cultured in Dulbecco’s modified eagle’s medium (DMEM) supplemented with 100 U/ml penicillin, 10% FBS, 100 mg/ml streptomycin, and 50 mM glutamine. Subsequently, the cells were detached using trypsin and seeded into a 96-well cell culture plate for the MTT (3-(4,5-Dimethylthiazol-2-yl)-2,5-diphenyltetrazolium bromide) assay. The test compound was dissolved in cell culture grade DMSO. Upon reaching 70% confluency, the cells in the 96-well plates were treated with the compound at five concentrations (0.01, 0.1, 1, 10, 100 µM) and maintained at 37 °C for 48 h in DMEM supplemented with 1% antibiotic and 10% FBS. After 48 h, the cells were washed with PBS and then incubated with 100 µl of fresh media in each well, along with 10 µl of MTT reagent (5 mg/ml), for an additional 4 h. Following this, the media was removed, and 100 µl of cell culture grade DMSO was added to dissolve the formazan crystals formed by the reduction of MTT by live cells. The quantity of formazan crystals formed was determined by measuring the absorbance at 570 nm wavelength using an ELISA plate reader (Bio-Tek). Each test was conducted in triplicate^52^.

The cell toxicity (%) was calculated using the following formula:

$$\% \text{Cell toxicity}=100-\frac{\text{Absorbance of treated well}}{\text{Absorbance of untreated well}} \times 100.$$

### Statistical analysis

The experiments were performed in triplicate, and the results were displayed as mean ± SD (Standard Deviation). The Microsoft Office Professional Plus 2013 (Excel) was utilized for graph plotting and calculation of SD and other binding parameters.

## Results and discussion

### Molecular docking studies

Computer-aided drug designing especially molecular docking and molecular dynamic simulation methods have proven to be an alternative approach to screening potential drug candidates against specific diseases in relatively less time. Molecular docking studies were performed to get an insight into the binding efficiency of novel azo-anchored 3,4-dihydroimidazo[4,5-*b*]indole derivatives **5*(a–r)*** as inhibitors against the main protease (M^pro^) of SARS-CoV-2 (PDB ID: 6LU7). On the basis of inference gathered from the literature, the main protease (M^pro^) of SARS-CoV-2 has an active site between domain I and domain II of protein 6LU7. The catalytic dyad (Cys145-His41) is present on the active site of the protein and both domains contribute one residue to the catalytic dyad ^6,37,53–56^. The Cys-His-catalytic dyad of M^pro^ exhibits protease activity^19^. Thus, the inhibition of the catalytic dyad in the main protease (M^pro^) could become an alluring target for designing and screening an effective drug for coronavirus^6,37^.

The computer-aided drug design methods afford an alternative way to screen the potential drug candidates for the treatment of specific diseases in relatively less time^57,58^. Therefore, in the present work, the molecular docking methods were performed to study the efficiency of novel azo-anchored 3,4-dihydroimidazo[4,5-*b*]indole derivatives **5*(a–r)*** as inhibitors against the main protease (M^pro^) of SARS-CoV-2. The results obtained from docking indicate the strong interaction of azo-anchored 3,4-dihydroimidazo[4,5-*b*]indole derivatives **5*(a–r)***, native ligand, and the standard drugs with the main protease (M^pro^) of SARS-CoV-2 near the cleft between domain I and domain II. A summary of the docking results of synthesized derivatives **5*(a–r)*** in comparison with native ligand and standard drugs with main protease (M^pro^) are summarized in Table 1.

**Table 1:** The Docking scores of azo-anchored 3,4-dihydroimidazo[4,5-*b*]indole derivatives **5*(a–r)***, native ligand, and standard drugs docked with the main protease protein (M^pro^) target selected for screening.

Compound name	MolDock score	Rerank score (kJ/mol)	Interaction energy (kJ/mol)	Steric	HBond (kJ/mol)
***5(a)***	− 141.078	− 87.044	− 155.441	− 154.658	− 0.783
***5(b)***	− 157.749	− 116.360	− 169.413	− 169.355	− 0.059
***5(c)***	− 164.168	− 116.618	− 169.873	− 169.786	− 0.087
***5(d)***	− 160.898	− 44.491	− 165.504	− 161.747	− 3.757
***5(e)***	− 160.346	− 65.514	− 169.517	− 164.643	− 4.874
***5(f)***	− 153.076	− 96.687	− 172.143	− 167.445	− 4.698
***5(g)***	− 166.206	− 118.948	− 168.116	− 163.664	− 4.452
***5(h)***	− 154.077	− 112.022	− 165.373	− 162.882	− 2.491
***5(i)***	− 167.384	− 120.308	− 174.048	− 173.829	− 0.219
***5(j)***	− 168.761	− 121.115	− 174.364	− 167.354	− 7.010
***5(k)***	− 158.884	− 87.418	− 162.866	− 160.398	− 2.468
***5(l)***	− 163.826	− 98.627	− 172.493	− 166.874	− 5.619
***5(m)***	− 165.156	− 92.563	− 169.989	− 161.903	− 8.086
***5(n)***	− 147.505	− 105.140	− 160.303	− 159.982	− 0.321
***5(o)***	− 153.854	− 93.671	− 158.746	− 158.728	− 0.018
***5(p)***	− 156.873	− 115.578	− 160.984	− 159.013	− 1.972
***5(q)***	− 137.976	− 76.030	− 163.179	− 162.678	− 0.501
***5(r)***	− 159.665	− 93.689	− 169.601	− 167.119	− 2.482
***N3***	− 164.767	− 49.350	− 204.289	− 200.365	− 3.924
***Remdesivir***	− 168.563	− 121.734	− 163.008	− 155.168	− 7.841
***Paxlovid***	− 158.512	− 32.757	− 144.348	− 133.162	− 11.187
***Molnupiravir***	− 117.187	− 101.975	− 132.411	− 127.359	− 5.052
***Chloroquine***	− 121.374	− 94.560	− 139.228	− 136.055	− 3.173
***Hydroxychloroquine***	− 133.871	− 104.424	− 151.879	− 149.218	− 2.600

### Visualization of docking results

**Figure 4:**
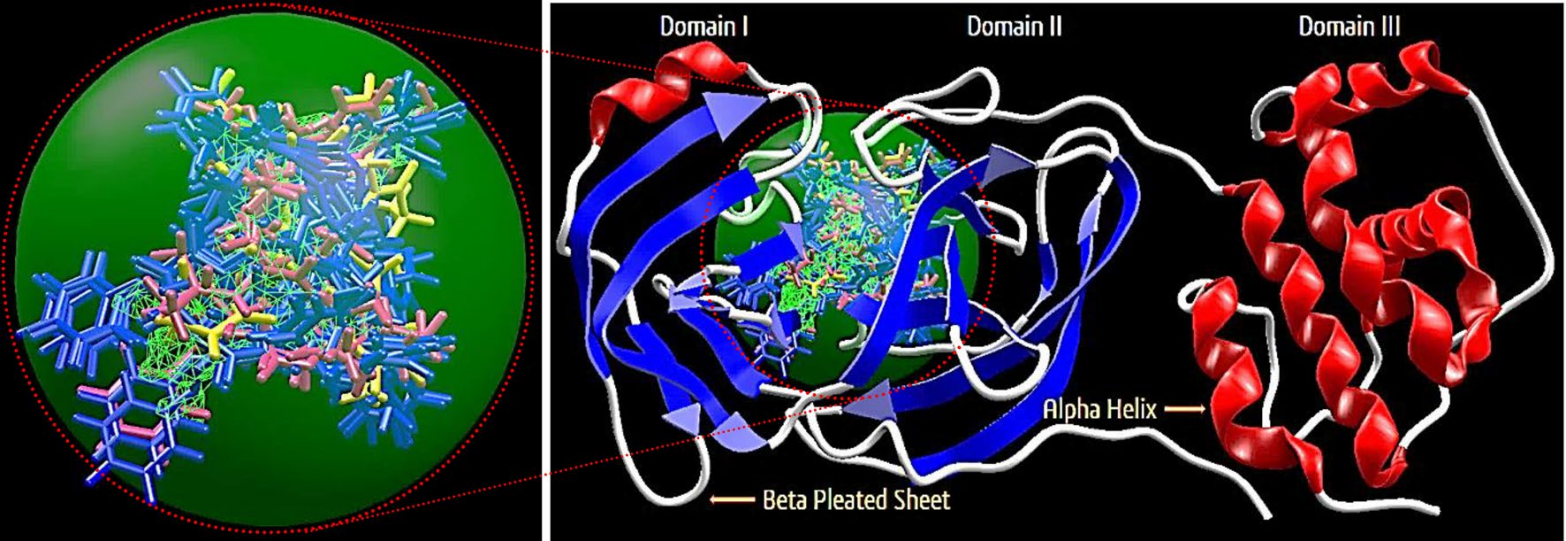
The Secondary structure of the main protease protein (M^pro^) of SARS-CoV-2 with the domain I, II, and III (Red circle represents the catalytically active site of M^pro^) bind with azo-anchored 3,4-dihydroimidazo[4,5-*b*] indole derivatives **5*(a–r)*** (Blue Colour), inhibitor (N3) (Yellow Colour) and Standard Drugs (Pink Colour) inside the cavity (green framework).

On the basis of inference gathered from the literature, the amino acid residues His 41, Cyd 145, and Glu 166 are significant residues in the substrate-binding site, and the contribution of these residues in the formation of hydrogen bonds could be prominent for the inhibitory effect of main protease (M^pro^)^6,37,59^. The secondary structure of the main protease (M^pro^) of the SARS-CoV-2 target with the detected active site between domain I and domain II is presented in Fig. 4. The results obtained from the docking studies prove that all the synthesized derivatives **5*(a–r)***, native ligand, and all the standard drugs fit inside the core pocket region of the protease at the interface between domain I and domain II. The screening results against the main protease (M^pro^) target showed that compound **5*(j)*** exhibited the highest MolDock score (**− 168.761**) among the series and in comparison with native ligand and standard drugs.

The visualization of the docking result of ligand **5*(j)*** with main protease (6LU7) revealed that the **5*(j)*** binds most closely with the Protein at the interface between domain I and domain II with the highest moldock score of − **168.761,** followed by standard drug Remdesivir. The significant binding of protease protein is alleviated by a network of five hydrogen bonds formed by the amino acids of the target protein. The nitrogen atom in the indole ring of **5*(j)*** shows hydrogen bond interaction with the carbonyl group of the amino acid residue His 164 at a distance of 3.58094 Å. The NH_2_ group of the amino acid residue Gly 143 exhibits two hydrogen bond interactions with the nitrogen atom and oxygen atom of the carbonyl group attached to the nitro group of the respective ligand. The two hydrogen bond interactions are evinced between the oxygen atom of a hydroxyl group attached to the nitro group of **5*(j)*** with the nitrogen atom of the NH_2_ group of the amino acid residues of Cys 145 and Ser 144, respectively. The molecular interactions analyses of azo-anchored 3,4-dihydroimidazo[4,5-*b*] indole derivatives **5*(a–r)***, native ligand, and standard drug with main protease (6LU7) of novel coronavirus (COVID-19) are summarized in Table 2.

**Table 2:** Molecular interactions analyses of azo-anchored 3,4-dihydroimidazo[4,5-*b*]indole derivatives **5*(a–r)***, native ligand and standard drug with main protease (6LU7) of novel coronavirus (COVID-19).

Compound name	Interaction	Bond energy (kJ/mol)	Bond length (Å)
**5*(a)***	Glu 166 (N)–N (7)	− 0.78322	3.25512
**5*(b)***	Arg 188 (N)–N (1)	− 0.05882	3.45467
**5*(c)***	Arg 188 (N)–N (1)	− 0.08748	3.46560
**5*(d)***	Gly 143 (N)–N (1)	− 1.77979	3.04126
	Asn 142 (N)–N (20)	− 1.97707	2.69434
**5*(e)***	Gly 143 (N)–N (1)	− 1.35505	2.99726
	Ser 144 (N) –N (1)	− 1.11068	3.15174
	Cys 145 (N)–N (1)	− 1.21288	3.28022
	Asn 142 (N)–O (20)	− 1.19562	2.52404
**5*(f)***	Gly 143 (N)–N (1)	− 1.71014	2.94426
	Ser 144 (N)–N (1)	− 0.53364	3.38661
	Cys 145 (N)–N (1)	− 0.28868	3.53197
	Leu 141 (O)–O (20)	− 1.20936	3.35813
	His 163 (N)–O (20)	− 2.50000	2.67386
**5*(g)***	Glu 166 (N)–N (1)	− 1.32885	3.28700
	Gly 143 (N)–N (11)	− 0.24474	3.50152
	Gly 143 (N)–N (12)	− 0.55322	3.18427
	Ser 144 (N)–O (21)	− 0.09579	3.55401
	Cys 145 (N)–O (21)	− 0.56244	2.98818
	Cys 145 (S)–O (22)	− 0.81895	3.43621
	His 163 (N)–O (22)	− 1.32771	3.33446
	His 164 (O)–O (22)	− 1.80757	2.60027
**5*(h)***	Arg 188 (O)–N (3)	− 2.49108	2.59893
**5*(i)***	Arg 188 (O)–N (1)	− 0.21934	3.35917
**5*(j)***	His 164 (O)–N (3)	− 0.03408	3.58094
	Gly 143 (N)–N (20)	− 1.84517	3.17214
	Gly 143 (N)–O (21)	− 2.50000	2.63811
	Ser 144 (N)–O (22)	− 0.74367	2.86896
	Cys 145 (N)–O (22)	− 1.88696	2.76371
**5*(k)***	Gly 143 (N)–N (1)	− 2.46818	3.09523
**5*(l)***	Gly 143 (N)–N (1)	− 1.48450	3.02026
	Ser 144 (N)–N (1)	− 0.85707	3.26919
	Cys 145 (N)–N (1)	− 0.77722	3.39796
	Glu 166 (O)–O (20)	− 2.50000	3.09933
**5*(m)***	Glu 166 (N)–N (1)	− 1.03566	3.38715
	Gly 143 (N)–O (22)	− 2.19064	3.16187
	Leu 141 (O)–O (21)	− 2.50000	2.60112
	Ser 144 (N)–O (21)	− 1.57214	2.91046
	Cys 145 (N)–O (21)	− 0.78721	3.18642
**5*(n)***	Asn 142 (N)–N (12)	− 0.32097	3.49473
**5*(o)***	Asp 187 (O)–N (3)	− 0.01794	3.58792
**5*(p)***	Glu 166 (N)–N (1)	− 0.90582	3.41884
	Gly 143 (N)–N (11)	− 1.06588	3.33714
**5*(q)***	Glu 166 (N)–N (7)	− 0.50059	3.30991
**5*(r)***	Leu 141 (O)–O (22)	− 2.50000	2.65716
	His 163 (N)–O (22)	− 2.50000	3.09564
***N3***	Asn 142 (N)–O (3)	− 0.94711	3.13165
	Gln 189 (O)–O (25)	− 1.45527	3.30895
	Glu 166 (N)–O (26)	− 0.81058	3.40707
	Cys 145 (S)–O (28ʹ)	− 0.71055	3.45789
***Remdesivir***	Asn 142 (N)–O (26)	− 0.09860	3.52228
	Leu 141 (O)–O (27)	− 2.50000	2.62061
	His 163 (N)–O (27)	− 2.46652	3.10670
	His 163 (O)–O (28)	− 1.62613	2.11300
	Gly 143 (N)–O (2)	− 2.50000	2.84441
	Gln 189 (N)–O (14)	− 0.65971	3.28812
	Asn 142 (N)–O (16)	− 1.24203	3.35159
***Paxlovid***	His 164 (O)–N (20ʹ)	− 1.35520	3.32896
	Glu 166 (N)–O (24ʹ)	− 1.40353	2.65768
	Cys 145 (S)–O (8)	− 2.50000	2.97352
	His 164 (O)–N (9)	− 0.03638	3.44101
	Ser 144 (N)–N (11ʹ)	− 1.13127	2.62396
	Cys 145 (O)–N (11ʹ)	− 2.00936	3.13802
	Phe 140 (O)–N (17)	− 0.04508	3.08078
	Glu 166 (O)–N (17)	− 2.33711	2.89959
	Gly 143 (N)–N (11ʹ)	− 0.36884	2.48744
***Molnupiravir***	Gln 192 (O)–O (6)	− 2.50000	3.09814
	Tyr 54 (O)–O (10ʹ)	− 2.50000	2.83582
	Gln 189 (O)–O (11ʹ)	− 0.03730	2.85621
	Met 49 (O)–O (11ʹ)	− 1.87793	3.22441
	Asp 187 (O)–O (10ʹ)	− 1.21826	3.35635
***Chloroquine***	Gly 143 (N)–N (7)	− 0.61675	3.33340
	Ser 144 (N)–N (7)	− 1.11225	3.13571
	Cys 145 (N)–N (7)	− 1.44394	3.07378
***Hydroxychloroquine***	Arg 188 (N)–N (7)	− 0.16032	3.04526
	Phe 140 (O)–O (22)	− 2.50000	2.90046
	Glu 166 (O)–O (22)	− 2.46540	3.10692

**Figure 5:**
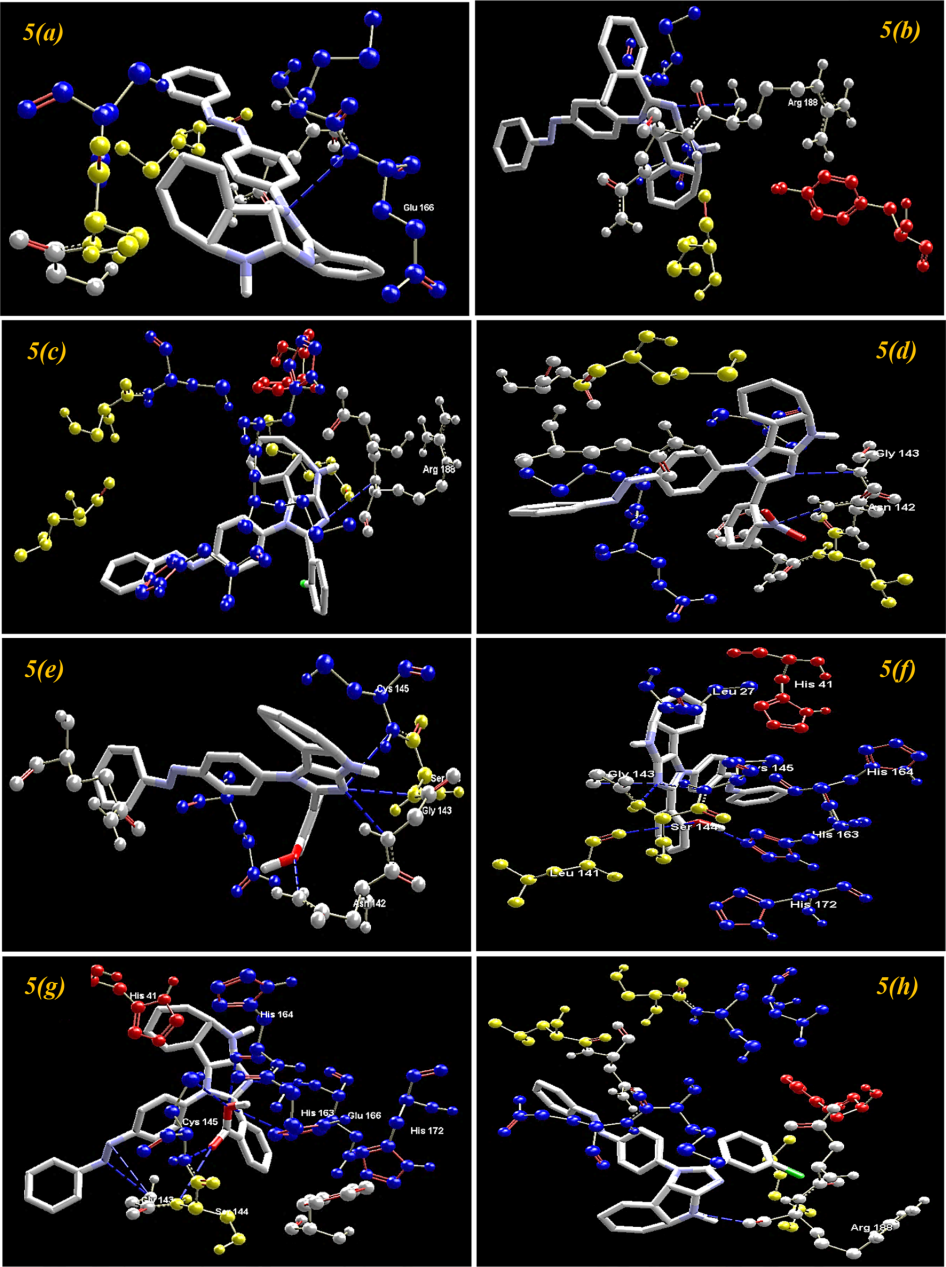

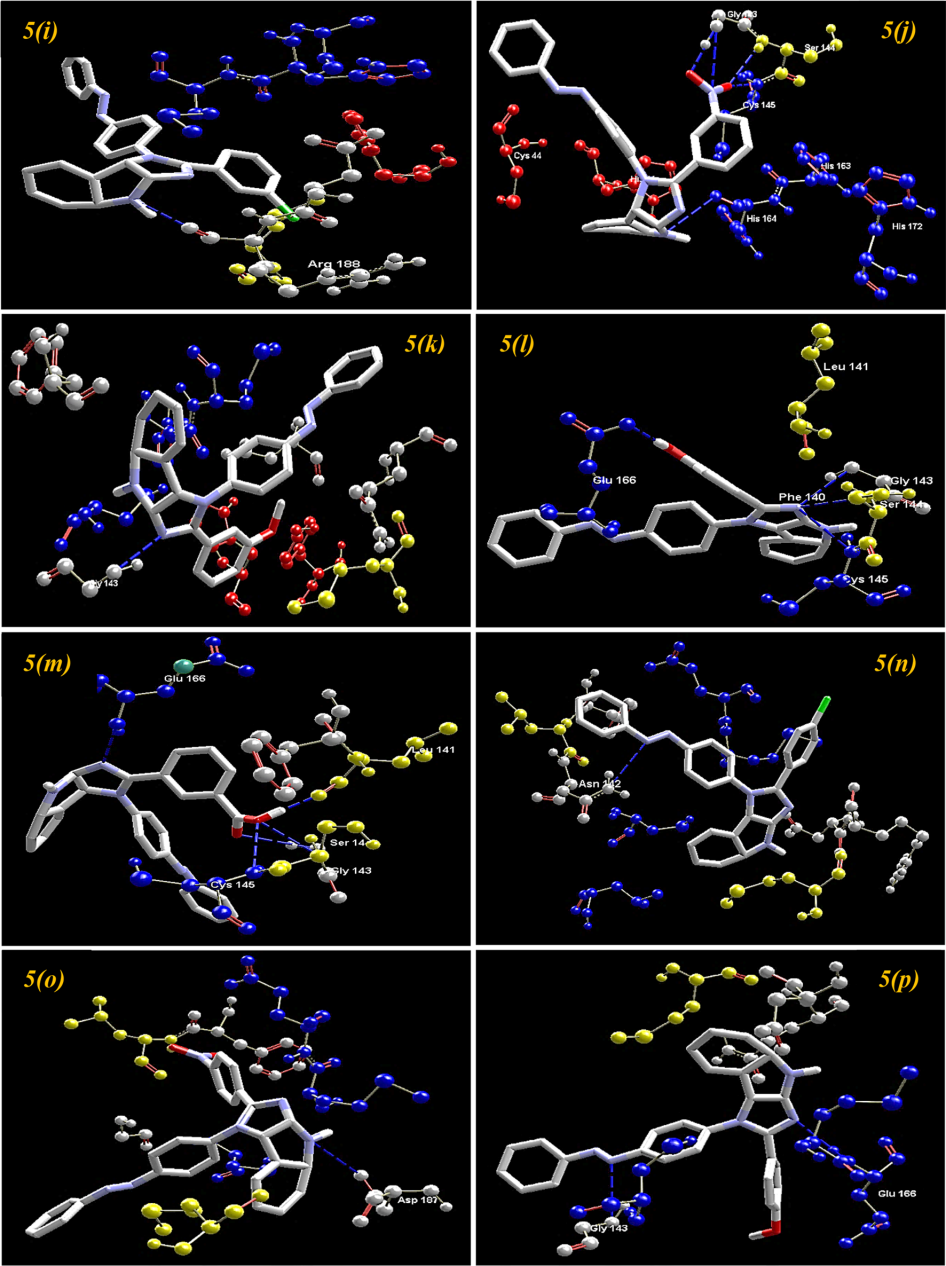

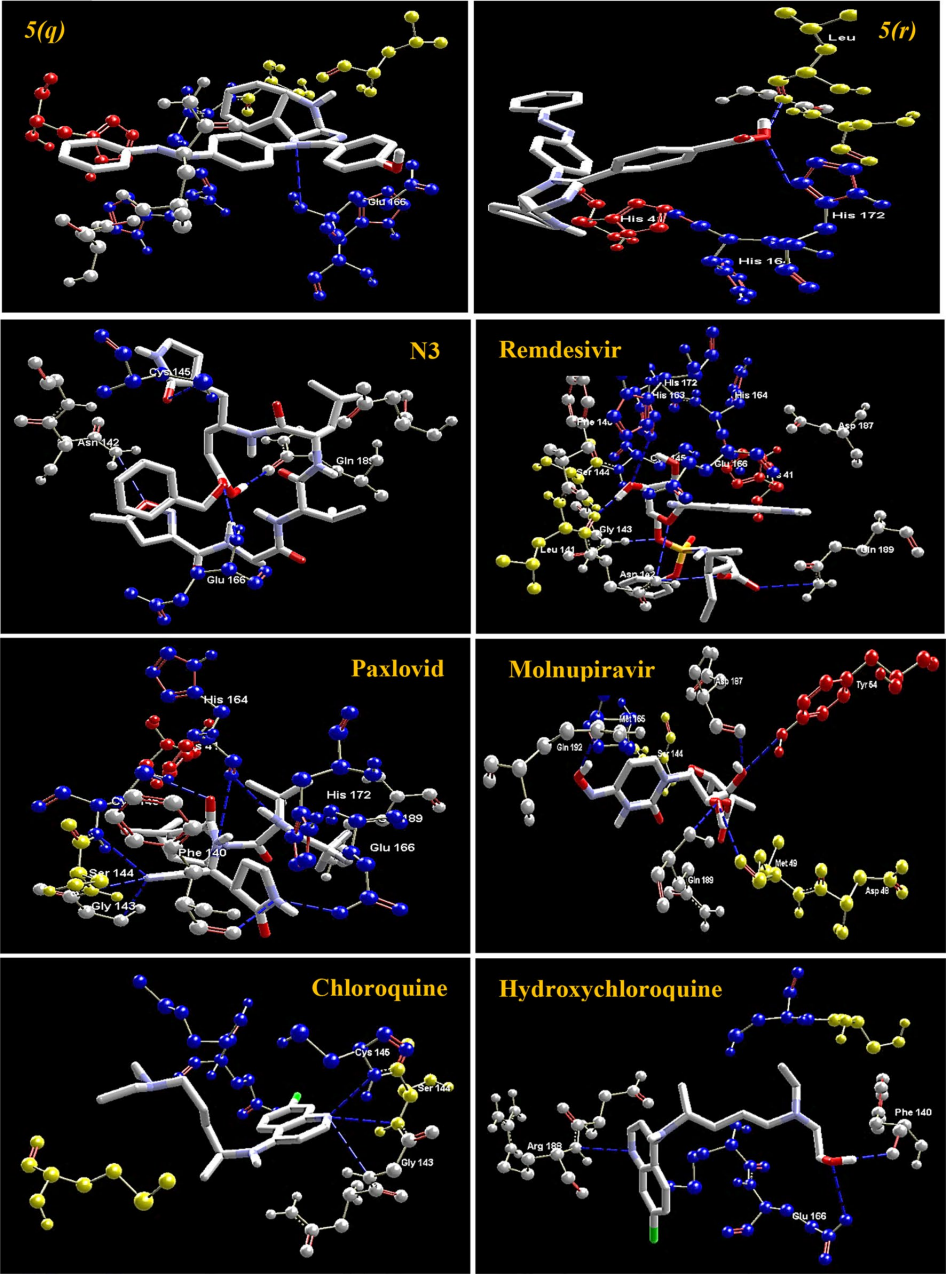
The hydrogen-bond interaction of azo-anchored 3,4-dihydroimidazo[4,5-*b*]indole derivatives **5*(a–r)****,* inhibitor (N3), and standard drug with main protease protein (M^pro^) of SARS-CoV-2.

On comparing with the inhibitor (N3) of protease protein (M^pro^)^60–62^, the synthesized compounds **5*(g)***, **5*(i)****,* and **5*(m)*** demonstrate superior binding affinities with moldock scores of − 166.206, − 167.384, and − 165.156, respectively. The screening results revealed that all the synthesized ligands **5*(a–r)*** show enhanced binding affinities in comparison with the Molnupiravir, Chloroquine, and Hydroxychloroquine against protease protein (M^pro^) with the least moldock score of − 117.187, − 121.374, and − 133.871, respectively. When compared with the other standard drug Paxlovid, ten azo-anchored derivatives demonstrate better binding affinities score higher than − 158.512. The results revealed that the substitution of electron-withdrawing groups on the aromatic ring enhances the binding affinity between ligand and protein. The substituent of the varied groups at *ortho*, *meta,* and *para* positions in the aromatic ring of ligands also indicates that the binding affinities can further be enhanced by the substitution at the *meta* position, as compared with the *ortho* followed by *para* positions. The Hydrogen-bond interaction of azo-anchored 3,4-dihydroimidazo[4,5-*b*]indole derivatives **5*(a–r)****,* inhibitor (N3), and standard drug with main protease protein (M^pro^) of SARS-CoV-2 are shown in Fig. 5.

### In vitro cytotoxicity assay

**Figure 6:**
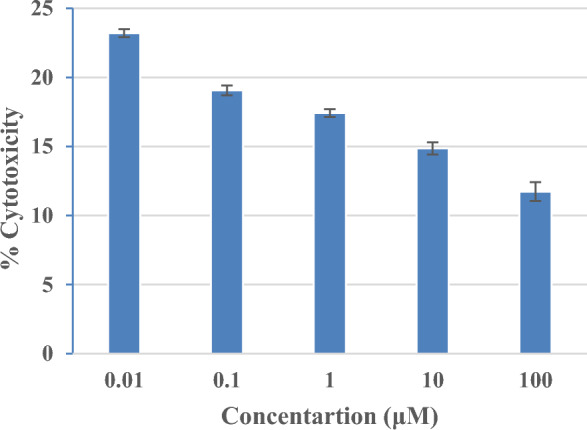
Cytotoxicity of synthesized compound **5*(j)*** against human normal cell line HEK293.

To assess its safety profile, the synthesized compound **5*(j)***, identified as having the highest binding affinity through molecular docking studies, underwent evaluation against Human Embryonic Kidney cells (HEK293) using the MTT assay. The results revealed negligible toxicity of compound **5*(j)*** across a range of concentrations (10^–4^ to 10^–8^ M), indicating promising potential for in vivo application. The Cytotoxicity profile of compound **5*(j)*** against human normal cell line HEK293 is depicted in Fig. 6. Specifically, treatment with compound **5*(j)*** resulted in HEK293 cell survivals exceeding 77%, 81%, 83%, 86%, and 89% at concentrations of 10^–4^, 10^–5^, 10^–6^, 10^–7^, and 10^–8^ M, respectively. These findings unequivocally demonstrate that the tested compound remained well within acceptable toxicity thresholds, underscoring their favourable safety profile and strong candidacy for further in vivo investigations.

### In silico prediction of drug-likeness properties

In order to determine the drug concentrations in the assorted parts of the organism with respect to time, the pharmacokinetic parameters are premeditated. When drugs are administered into the body of an organism, they need to cross diverse biological barriers. Thus, the main properties like absorption, distribution, metabolism, and excretion (ADME) are very significant parameters for any compound to be considered as a drug^6,63,64^. Therefore, prior to any clinical and animal studies it is reasonably significant to ascertain the pharmacokinetic properties of the synthesized molecules under consideration.

The Pfizer’s rule of five also recognized as Lipinski’s rule of five (5) is a thumb rule for evaluating the drug-likeness and determining if an inhibitor with certain pharmacological and biological assets would be an orally active drug in the human body^65,66^. The rule states that a molecule or an inhibitor can be considered biologically active for oral administration in humans if it must not violate more than one of these thresholds; the molecular weight (Mw) of the molecule must be < 500 Daltons, octanol/water partition coefficient (iLOGP) must be ≤ 5, number of hydrogen bond acceptors (nHBA) must be ≤ 10, number of hydrogen bond donors (nHBD) ≤ 5, and topological polar surface area (TPSA) ≤ 40 Å^2^). The physicochemical parameters are associated with acceptable aqueous solubility and intestinal permeability and encompass the first step of oral bioavailability. To ascertain the drug-likeness character of compounds, a computer-aided online Swiss ADME database is used for determining the substantial pharmacokinetic properties^67^.

The outputs of some ADME and drug-likeness properties of synthesized derivatives **5*(a–r)*** in comparison with inhibitor (N3) and standard drugs are summarized in Table 3. The results reveal that the synthesized azo-anchored 3,4-dihydroimidazo[4,5-*b*]indole derivatives **5*(a–r)*** have zero violation of Lipinski’s rule and good pharmacokinetic properties embracing low gastrointestinal absorption, non-substrate to P-glycoprotein, not possess Blood–brain barrier (BBB) permeant and CYP1A2 inhibitor. While the inhibitor (N3) of the main protease protein (M^pro^), and Remdesivir have comparatively low bioavailability of 17% as compared to all the synthesized ligands **5*(a–r)***. The standard drugs Paxlovid, Chloroquine, and Hydrochloroquine comprise high gastrointestinal absorption as compared with all the synthesized ligands **5*(a–r)****,* native ligand, Remdesivir, and Molnupiravir.

**Table 3:** The Lipinski’s properties and pharmacokinetic properties (ADME) of the synthesized azo-anchored 3,4-dihydroimidazo[4,5-*b*] indole derivatives **5*(a–r)***, native ligand and standard drugs.

Compound name	SA	GI	BBB	Pgp	BS	MW(g/mol)	nHBA	nHBD	TPSA (Å)	Log *P*_o/w_	Log S	CYP1A2 inhibitor	nLV
*5(a)*	3.18	Low	No	No	0.55	413.47	3	1	58.33	5.90	− 8.04 (PS)	No	0
*5(b)*	3.36	Low	No	No	0.55	427.50	3	1	58.33	6.19	− 8.41 (PS)	No	0
*5(c)*	3.23	Low	No	No	0.55	447.92	3	1	58.33	6.36	− 8.68 (PS)	No	0
*5(d)*	3.53	Low	No	No	0.55	458.47	5	1	104.15	5.24	− 8.82 (PS)	No	0
*5(e)*	3.31	Low	No	No	0.55	443.50	4	1	67.56	5.85	− 8.20 (PS)	No	0
*5(f)*	3.22	Low	No	No	0.55	429.47	4	2	78.56	5.48	− 8.09 (PS)	No	0
*5(g)*	3.36	Low	No	No	0.56	457.48	5	2	95.63	5.43	− 8.33 (PS)	No	0
*5(h)*	3.19	Low	No	No	0.55	447.92	3	1	58.33	6.40	− 8.68 (PS)	No	0
*5(i)*	3.37	Low	No	No	0.55	427.50	3	1	58.33	6.20	− 8.41 (PS)	No	0
*5(j)*	3.43	Low	No	No	0.55	458.47	5	1	104.15	5.29	− 8.82 (PS)	No	0
*5(k)*	3.29	Low	No	No	0.55	443.50	4	1	67.56	5.88	− 8.20 (PS)	No	0
*5(l)*	3.19	Low	No	No	0.55	429.47	4	2	78.56	5.45	− 8.09 (PS)	No	0
*5(m)*	3.40	Low	No	No	0.56	457.48	5	2	95.63	5.46	− 8.33 (PS)	No	0
*5(n)*	3.19	Low	No	No	0.55	447.92	3	1	58.33	6.40	− 8.68 (PS)	No	0
*5(o)*	3.36	Low	No	No	0.55	458.47	5	1	104.15	5.27	− 8.82 (PS)	No	0
*5(p)*	3.26	Low	No	No	0.55	443.50	4	1	67.56	5.87	− 8.20 (PS)	No	0
*5(q)*	3.17	Low	No	No	0.55	429.47	4	2	78.56	5.48	− 8.09 (PS)	No	0
*5(r)*	3.27	Low	No	No	0.56	457.48	5	2	95.63	5.43	− 8.33 (PS)	No	0
*N3*	6.43	Low	No	Yes	0.17	680.79	9	5	197.83	2.69	− 7.18 (PS)	No	0
*Remdesivir*	6.33	Low	No	Yes	0.17	602.58	12	4	213.36	1.49	− 6.01 (PS)	No	0
*Paxlovid*	4.82	High	No	Yes	0.55	499.53	8	3	131.40	1.87	− 4.56 (MS)	No	0
*Molnupiravir*	4.65	Low	No	Yes	0.55	329.31	8	4	146.37	− 0.63	− 1.94 (VS)	No	0
*Chloroquine*	2.76	High	Yes	No	0.55	319.87	2	1	28.16	4.15	− 4.95 (MS)	Yes	0
*Hydroxychloroquine*	2.82	High	Yes	No	0.55	335.87	3	2	48.39	3.29	− 4.28 (MS)	Yes	0

*SA* synthetic accessibility, *GI* gastrointestinal absorption, *BBB* blood–brain barrier permeant, *Pgp* P-glycoprotein substrate, *BS* bioavailability score, *MW* molecular weight, *nHBD* number of hydrogen bond donor, *nHBA* number of hydrogen bond acceptor, *TPSA* topological polar surface area, *Log Po/w* octanol/water partition coefficient, *Log S* water-soluble capability, *CYP* cytochrome P450, *nLV* number of Lipinski violation, *MS* moderately soluble, *PS* poorly soluble, *VS* very soluble.

The azo-anchored 3,4-dihydroimidazo[4,5-*b*]indole derivatives **5*(a–r)*** have a synthetic accessibility score lower than native ligand, Remdesivir, Paxlovid, and Molnupiravir in the range of 3.17–3.53 and higher than chloroquine, and Hydroxychloroquine. The compounds with high SA scores are generally difficult to synthesize, whereas the compounds with low SA score values are easily synthetically accessible. The low SA score indicates that all the synthesized derivatives **5*(a–r)*** are suited for biological screening activities. All the synthesized derivatives **5*(a–r)*** with a bioavailability of about 55–56% have a consensus lipophilicity (Log *P*_o/w_) value in the range of 5.24–6.40. The high and positive lipophilicity values indicate that all the compounds are more lipophilic and have the tendency to be extensively bound to the plasma protein^68^. Therefore, drug molecules are requisite to possess lipophilicity to achieve good absorption in the body. However, the synthesized compounds **5*(a–r)*** are poorly soluble in water as signified by their solubility values (LogS) in the range from − 8.04 to − 8.82.

**Figure 7:**
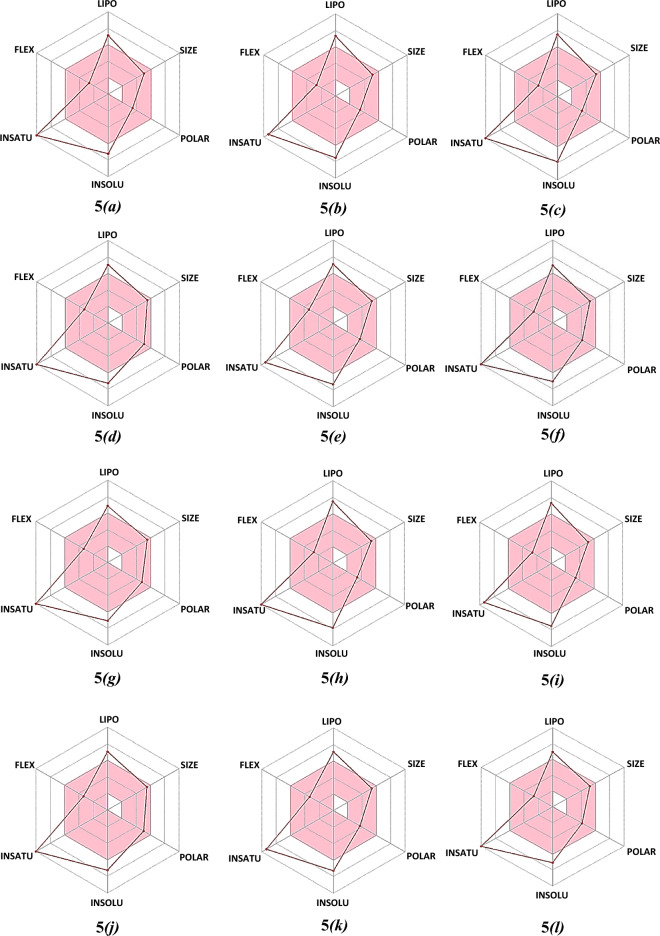

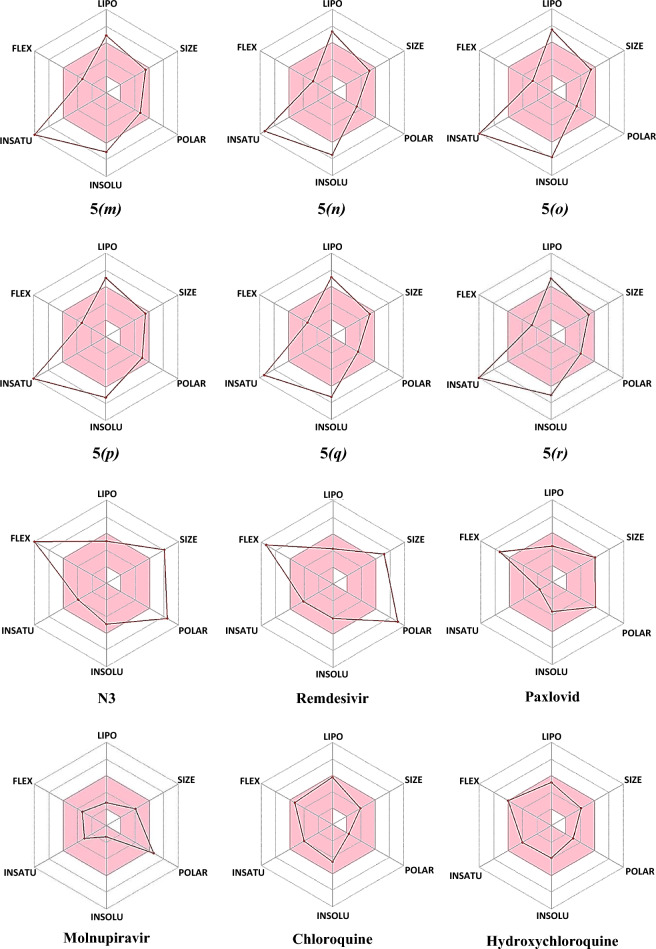
The bioavailability radars of azo-anchored 3,4-dihydroimidazo[4,5-*b*]indole derivatives **5*(a–r)***, the native ligand of protease protein (M^pro^), and standard drug.

Furthermore, the bioavailability radar delineates a rapid appraisal of the drug-likeness of a molecule by intriguing six physicochemical properties are taken into consideration viz*.* Lipophilicity, Size, Polarity, Solubility, Saturation, and Flexibility^69–71^. The bioavailability radars of azo-anchored 3,4-dihydroimidazo[4,5-*b*]indole derivatives **5*(a–r)***, the native ligand of protease protein (M^pro^), and standard drugs are illustrated in Fig. 7. The bioavailability radar demonstrates that all the synthesized compounds **5*(a–r)*** are depicted in the pink area of the radar plot. Thus, all the compounds are predicted to be orally bioavailable with low flexibility, moderate solubility, high polarity, less toxicity, and good absorption. The analyses of pharmacokinetic results reveal that the azo-anchored 3,4-dihydroimidazo[4,5-*b*]indole derivatives **5*(a–r)*** qualify the criteria of drug-likeness with no violation of the Lipinski rule. Thus, it is inferred that these compounds can serve as potential drug candidates to combat the disease.

## Conclusion

The synthesis of pharmacologically significant compounds through ultrasonically mediated sustainable methods represents a significant advancement in medicinal chemistry, especially within the framework of environmental consciousness. Our exploration into the virtual screening of ligands **5*(a–r)*** against the main protease (M^pro^) of SARS-CoV-2 has revealed substantial interactions with the active site, particularly at the interface between domain I and domain II of the targeted protein. The ligand **5*(j)*** has come to the limelight with superior binding affinity among the series and as compared with standard drugs and the native ligand of the main protease. This finding not only underscores the potency of our synthesized compounds but also explodes optimism for their pivotal role in combating viral infections.

Furthermore, the pharmacokinetic analyses have provided compelling evidence of the drug candidacy of these compounds. Their strict adherence to Lipinski’s rule and favourable pharmacokinetic attributes, including low gastrointestinal absorption, resistance to P-glycoprotein substrate status, and the inability to breach the Blood–Brain Barrier or inhibit CYP1A2, firmly establish them as promising drug candidates with impeccable safety profiles. The bioavailability radar further corroborates their suitability for oral administration, accentuating attributes of low flexibility, moderate solubility, high polarity, and minimal toxicity.

These attributes not only augment their therapeutic efficacy but also streamline their journey toward clinical translation, offering a beacon of hope in the ongoing battle against SARS-CoV-2 and future viral threats. The present study paves the way for in vitro and in vivo testing of azo-imidazole scaffolds, not only to halt the spread of the virus but also to herald a new era of antiviral therapeutics. Through this sustainable synthesis and pioneering drug discovery, we are on the verge of catalyzing significant advancements in medicinal chemistry, driving humanity towards a healthier, more resilient future.

### Supplementary Information

Supplementary Information.

## Data Availability

All data generated or analysed during this study are included in this published article and its Supplementary Information file.
