# Supplementary material for: Synthesis and in silico inhibitory action studies of azo-anchored imidazo[4,5-b]indole scaffolds against the COVID-19 main protease (Mpro)
Source: Sci Rep. 2024 May 6;14:10419. doi: 10.1038/s41598-024-57795-4 (PMC11074333; doi:10.1038/s41598-024-57795-4)
Supplement: Supplementary file 1 — Supplementary Information. [file 41598_2024_57795_MOESM1_ESM.docx]

**Synthesis and *In Silico* Inhibitory Action Studies of Azo-Anchored Imidazo[4,5-*b*]indole Scaffolds Against the COVID-19 Main Protease (M^pro^)**

Deepika Geedkar, Ashok Kumar, Pratibha Sharma*

*School of Chemical Sciences, Devi Ahilya University, Indore, Madhya Pradesh, India*

# *** Corresponding Author Email*: drpratibhasharma@yahoo.com*

***Table of Contents***

- General Information................................................................................................................02
- Physical analyses data for azo-anchored imidazo[4,5-*b*]indole derivatives *5(a-r) ...….…*03-20
- Figure S1-S30. The spectrums of azo-anchored imidazo[4,5-*b*]indole derivatives *5(a), 5(d),* and *5(h-o)*..........................................................................................................................21-49
- References...............................................................................................................................50
- **General Information**

All chemicals and solvents were purchased from Sigma Aldrich and Merck India. The reactions were performed in an aerobic atmosphere without any specific precautions. Melting points were determined in open capillary tubes on a Veego melting-point apparatus and were uncorrected. The ultrasound-promoted reactions were performed using a Cole Parmer-ultrasonic processor Model CPX 130, with a maximum power of 130 W, operating at an amplitude of 60% and a frequency of 20 kHz. All the products were characterized by their spectroscopic data (FTIR, ^1^H, and ^13^C NMR). The ^1^H and ^13^C NMR spectra of the synthesized compounds were recorded at 400 and 100 MHz, respectively, using Bruker Advance II 400 NMR spectrometer in CDCl_3_ solvent, and the chemical shifts (δ) were expressed in parts per million. Spin multiplicities are described as s (singlet), d (doublet), t (triplet), q (quartet), and m (multiplet). Fourier Transform Infra-Red (FTIR) spectra were recorded as ATR spectra within the range of 4000–400 cm^-1^ using Frontier Perkin-Elmer FTIR SP 10 STD. Thin-layer chromatography (TLC) is performed using precoated aluminum sheets with silica gel (Merck 60 F_254_). The mass spectra were attained on Water’s Q-Tof Micromass instrument using electron spray ionization (ESI) in positive mode.^1-4^

- *Physical analyses data for synthesized compounds*

S1. Compound *5(a)*: ***2-phenyl-1-(4-(phenyldiazenyl)phenyl)-1,4-dihydroimidazo[4,5-b]indole***

**

Compound 5*(a)* was prepared in 82% yield from *4-*amino-functionalized azo benzene (*1*) (1 mmol), benzaldehyde *2*(*a*) (1 mmol), indoline-2,3-dione (*3*) (1 mmol), ammonium acetate (*4*) (1 mmol) and (5 mol%) of L-proline in 10 ml of ethanol was irradiated through an ultrasonic wave at room temperature for the 18 minutes; Yellowish Solid; M.P.: 132-134°C;

**FTIR (ATR, υ, cm^−1^):** 3338 (N−H str.), 3087 (C−H, sp^2^), 2923 (C−H_Asym_, sp^3^), 2868 (C−H_Sym_, sp^3^), 1642 (C=C/C=N), 1589 (N=N), 1608, 1489, 1456 (CC ring str.);

^1^H NMR (400 MHz, CDCl_3_): δ_H_ ppm 7.33-7.46 (4H, m), 7.52-7.64 (5H, m), 7.72-7.83 (4H, m), 7.89-8.28 (5H, m), 9.96 (1H, s);

^13^C NMR (100 MHz, CDCl_3_): δ 111.3 (*C−5*), 114.4 (*C− 8, 8’*), 117.0 (*C−4’*), 118.3 (*C−5’*), 122.2 (*C−13,13’*), 124.2 (*C−9, 9’*), 125.7 (*C−16*), 127.1 (*C−17, 17’*), 127.8 (*C−15, 19*), 128.2 (*C−6, 14, 14’*), 128.4 (*C−6’, 18, 18’*), 132.9 (*C−7’*), 133.7 (*C−2’*), 136.3 (*C−4*), 144.0 (*C−1’*), 150.1 (*C−2*), 153.3 (*C−10, 12’*);

HRMS (ESI) *m/z*: 414.1605 [M+H]^+^;

Anal. Cald. for C_27_H_19_N_5_: C, 78.43; H, 4.63; N, 16.94 %. Found: C, 78.41; H, 4.65; N, 16.96 %.

**S2. Compound *5(b)*:** *1-(4-(phenyldiazenyl)phenyl)-2-o-tolyl-1,4-dihydroimidazo[4,5-b]indole*

**

Compound 5*(b)* was prepared in 86% yield from *4-*amino-functionalized azo benzene (*1*) (1 mmol), 2-methylbenzaldehyde *2*(*b*) (1 mmol), indoline-2,3-dione (*3*) (1 mmol), ammonium acetate (*4*) (1 mmol) and (5 mol%) of L-proline in 10 ml of ethanol was irradiated through an ultrasonic wave at room temperature for the 15 minutes; Yellowish Solid; M.P.: 139-141°C;

**FTIR (ATR, υ, cm^−1^):** 3334 (N−H str.), 3092 (C−H, sp^2^), 2928 (C−H_Asym_, sp^3^), 2854 (C−H_Sym_, sp^3^), 1638 (C=C/C=N), 1593 (N=N), 1613, 1498, 1462 (CC ring str.);

^1^H NMR (400 MHz, CDCl_3_): δ_H_ ppm 2.27 (3H, s), 7.12-7.37 (4H, m), 7.41-7.58 (4H, m), 7.76-7.88 (4H, m), 7.94-8.23 (5H, m), 9.95 (1H, s);

^13^C NMR (100 MHz, CDCl_3_): δ 20.0 (*C−20*), 111.3 (*C−5*), 114.4 (*C−8, 8’*), 117.0 (*C−4’*), 118.3 (*C−5’*), 120.4 (*C−16*), 122.2 (*C−13, 13’*), 124.2 (*C−9, 9’*), 124.5 (*C−17*), 127.8 (*C−15*), 128.2 (*C−6, 14, 14’*), 128.4 (*C−6’*, *18, 19*), 128.9 (*C−18’*), 132.9 (*C−7’*), 133.7 (*C−2’*), 136.3 (*C−4*), 136.4 (*C−17’*), 144.0 (*C−1’*), 150.1 (*C−4*), 153.3 (*C−10, 12’*);

HRMS (ESI) *m/z*: 428.5020 [M+H]^+^;

Anal. Cald. for C_28_H_21_N_5_: C, 78.67; H, 4.95; N, 16.38 %. Found: C, 78.69; H, 4.93; N, 16.41 %.

**S3. Compound *5(c)*:** *2-(2-chlorophenyl)-1-(4-(phenyldiazenyl)phenyl)-1,4-dihydroimidazo[4,5-b]indole*

Compound 5*(c)* was prepared in 89% yield from 4*-*amino-functionalized azo benzene (*1*) (1 mmol), 2-chlorobenzaldehyde *2*(*c*) (1 mmol), indoline-2,3-dione (*3*) (1 mmol), ammonium acetate (*4*) (1 mmol) and (5 mol%) of L-proline in 10 ml of ethanol was irradiated through an ultrasonic wave at room temperature for the 9 minutes; Yellowish Solid; M.P.: 134-136°C;

**FTIR (ATR, υ, cm^−1^):** 3332 (N−H str.), 3093 (C−H, sp^2^), 2927 (C−H_Asym_, sp^3^), 2873 (C−H_Sym_, sp^3^), 1648 (C=C/C=N), 1583 (N=N), 1614, 1495, 1469 (CC ring str.), 676 (C−Cl);

^1^H NMR (400 MHz, CDCl_3_): δ_H_ ppm 7.23-7.43 (4H, m), 7.49-7.62 (5H, m), 7.80-7.88 (4H, m), 7.92-8.28 (4H, m), 9.92 (1H, s);

^13^C NMR (100 MHz, CDCl_3_): δ 111.3 (*C−5*), 114.4 (*C−8, 8’*), 117.0 (*C−4’*), 118.3 (*C−5’*), 122.2 (*C−13,13’*), 124.2 (*C−9, 9’*), 127.8 (*C−15*), 128.2 (*C−6, 14, 14’*), 128.3 (*C−17, 19*), 128.4 (*C−6’, 18*), 128.7 (*C−18’*), 129.2 (*C−16*), 132.4 (*C−17’*), 132.9 (*C−7’*), 133.7 (*C−2’*), 136.3 (*C−4*), 144.0 (*C−1’*), 150.1 (*C−2*), 153.3 (*C−10, 12’*)

HRMS (ESI) *m/z*: 448.9232 [M+H]^+^;

Anal. Cald. for C_27_H_18_ClN_5_: C, 72.40; H, 4.05; N, 15.64 %. Found: C, 78.43; H, 4.03; N, 15.67 %.

**S4. Compound *5(d)*:** *2-(2-nitrophenyl)-1-(4-(phenyldiazenyl)phenyl)-1,4-dihydroimidazo[4,5-b]indole*

Compound 5*(d)* was prepared in 87% yield from 4*-*amino-functionalized azo benzene (*1*) (1 mmol), 2-nitrobenzaldehyde *2*(*d*) (1 mmol), indoline-2,3-dione (*3*) (1 mmol), ammonium acetate (*4*) (1 mmol) and (5 mol%) of L-proline in 10 ml of ethanol was irradiated through an ultrasonic wave at room temperature for the 8 minutes; Yellowish Solid; M.P.: 149-151°C;

**FTIR (ATR, υ, cm^−1^):** 3327 (N−H str.), 3095 (C−H, sp^2^), 2919 (C−H_Asym_, sp^3^), 2873 (C−H_Sym_, sp^3^), 1637 (C=C/C=N), 1591 (N=N), 1537 (N−O str.), 1616, 1487, 1473 (CC ring str.);

^1^H NMR (400 MHz, CDCl_3_): δ_H_ ppm 7.02-7.41 (4H, m), 7.49-7.63 (5H, m), 7.77-7.87 (4H, m), 7.90-8.05 (4H, m), 9.89 (1H, s);

^13^C NMR (100 MHz, CDCl_3_): δ 111.3 (*C−5*), 114.4 (*C−8, 8’*), 117.0 (*C−4’*), 118.3 (*C−5’*), 122.2 (*C−13,13’*), 124.2 (*C−9, 9’*), 127.8 (*C−15*), 128.2 (*C−6, 14, 14’*), 128.3 (*C−17, 19*), 128.4 (*C−6’, 18*), 128.7 (*C−18’*), 129.2 (*C−16*), 132.4 (*C−17’*), 132.9 (*C−7’*), 133.7 (*C−2’*), 136.3 (*C−4*), 144.0 (*C−1’*), 150.1 (*C−2*), 153.3 (*C−10, 12’*)

HRMS (ESI) *m/z*: 459.4729 [M+H]^+^;

Anal. Cald. for C_27_H_18_N_6_O_2_: C, 70.73; H, 3.96; N, 18.33 %. Found: C, 70.69; H, 3.98; N, 18.29 %.

**S5. Compound *5(e)*:** *2-(2-methoxyphenyl)-1-(4-(phenyldiazenyl)phenyl)-1,4-dihydroimidazo[4,5-b]indole*

Compound 5*(e)* was prepared in 83% yield from 4*-*amino-functionalized azo benzene (*1*) (1 mmol), 2-methoxybenzaldehyde *2*(*e*) (1 mmol), indoline-2,3-dione (*3*) (1 mmol), ammonium acetate (*4*) (1 mmol) and (5 mol%) of L-proline in 10 ml of ethanol was irradiated through an ultrasonic wave at room temperature for the 13 minutes; Yellowish Solid; M.P.: 154-156°C;

**FTIR (ATR, υ, cm^−1^):** 3329 (N−H str.), 3086 (C−H, sp^2^), 2948, 2935 (C−H_Asym_, sp^3^), 2871, 2858 (C−H_Sym_, sp^3^), 1645 (C=C/C=N), 1586 (N=N), 1608, 1492, 1457 (CC ring str.), 1043 (C−O);

^1^H NMR (400 MHz, CDCl_3_): δ_H_ ppm 3.96 (3H, s), 7.27-7.40 (4H, m), 7.48-7.59 (5H, m), 7.81-7.89 (4H, m), 7.97-8.18 (4H, m), 9.94 (1H, s);

^13^C NMR (100 MHz, CDCl_3_): δ 56.0 (*C−20’*), 111.3 (*C−5*), 114.4 (*C−8, 8’*), 115.0 (*C−16*), 115.8 (*C−18’*), 117.0 (*C−4’*), 118.3 (*C−5’*), 122.2 (*C−13, 13’*), 124.2 (*C−9, 9’*), 126.7 (*C−17*), 127.8 (*C−15*), 128.2 (*C−6, 14, 14’*), 128.4 (*C−6’, 18*), 129.4 (*C−19*), 132.9 (*C−7’*), 133.7 (*C−2’*), 136.3 (*C−4*), 144.0 (*C−1’*), 150.1 (*C−2*), 153.3 (*C−10, 12’*), 156.3 (*C−17’*);

HRMS (ESI) *m/z*: 443.5017 [M+H]^+^;

Anal. Cald. for C_28_H_21_N_5_O: C, 75.83; H, 4.77; N, 15.79 %. Found: C, 75.79; H, 4.80; N, 15.82 %.

**S6. Compound *5(f)*:** *2-(1-(4-(phenyldiazenyl)phenyl)-1,4-dihydroimidazo[4,5-b]indol-2-yl)phenol*

Compound *5(f)* was prepared in 87% yield from 4*-*amino-functionalized azo benzene (*1*) (1 mmol), 2-hydroxybenzaldehyde *2*(*f*) (1 mmol), indoline-2,3-dione (*3*) (1 mmol), ammonium acetate (*4*) (1 mmol) and (5 mol%) of L-proline in 10 ml of ethanol was irradiated through an ultrasonic wave at room temperature for the 18 minutes; Yellowish Solid; M.P.: 152-154°C;

**FTIR (ATR, υ, cm^−1^):** 3435 (O−H str.), 3334 (N−H str.), 3081 (C−H, sp^2^), 2941 (C−H_Asym_, sp^3^), 2865 (C−H_Sym_, sp^3^), 1649 (C=C/C=N), 1592 (N=N), 1611, 1497, 1464 (CC ring str.);

^1^H NMR (400 MHz, CDCl_3_): δ_H_ ppm 3.34 (1H, s), 7.23-7.42 (4H, m), 7.47-7.58 (5H, m), 7.76-7.87 (4H, m), 7.91-8.21 (4H, m), 9.92 (1H, s);

^13^C NMR (100 MHz, CDCl_3_): δ 111.3 (*C−5*), 114.4 (*C−8, 8’*), 115.0 (*C−16*), 116.8 (*C−18’*), 117.0 (*C−4’*), 118.3 (*C−5’*), 122.2 (*C−13, 13’*), 124.2 (*C−9, 9’*), 128.3 (*C−17*), 127.8 (*C−15*), 128.2 (*C−6, 14, 14’*), 128.4 (*C−6’, 18*), 129.4 (*C−19*), 132.9 (*C−7’*), 133.7 (*C−2’*), 136.3 (*C−4*), 144.0 (*C−1’*), 150.1 (*C−2*), 153.3 (*C−10, 12’*), 158.2 (*C−17’*):

HRMS (ESI) *m/z*: 430.46081 [M+H]^+^;

Anal. Cald. for C_27_H_19_N_5_O: C, 75.51; H, 4.46; N, 16.31 %. Found: C, 75.47; H, 4.48; N, 16.28 %.

**S7. Compound *5(g)*:** *2-(1-(4-(phenyldiazenyl)phenyl)-1,4-dihydroimidazo[4,5-b]indol-2-yl)benzoic acid*

Compound 5*(g)* was prepared in 84% yield from *4-*amino-functionalized azo benzene (*1*) (1 mmol), 2-carboxybenzaldehyde *2*(*g*) (1 mmol), indoline-2,3-dione (*3*) (1 mmol), ammonium acetate (*4*) (1 mmol) and (5 mol%) of L-proline in 10 ml of ethanol was irradiated through an ultrasonic wave at room temperature for the 16 minutes; Yellowish Solid; M.P.: 147-149°C;

**FTIR (ATR, υ, cm^−1^):** 3438 (O−H str.), 3331 (N−H str.), 3082 (C−H, sp^2^), 2927 (C−H_Asym_, sp^3^), 2873 (C−H_Sym_, sp^3^), 1637 (C=C/C=N), 1594 (N=N), 1605, 1496, 1462 (CC ring str.), 1047 (C−O);

^1^H NMR (400 MHz, CDCl_3_): δ_H_ ppm 5.39 (1H, s), 7.23-7.43 (4H, m), 7.49-7.62 (5H, m), 7.80-7.88 (4H, m), 7.92-8.38 (4H, m), 9.96 (1H, s);

^13^C NMR (100 MHz, CDCl_3_): δ 111.3 (*C−5*), 114.4 (*C−8, 8’*), 117.0 (*C−4’*), 118.3 (*C−5’*), 120.4 (*C−16*), 122.2 (*C−13,13’*), 123.9 (*C−17’*), 124.2 (*C−9, 9’*), 124.5 (*C−17*), 127.8 (*C−15*), 128.2 (*C−6, 14, 14’*), 128.4 (*C−6’, 18, 19*), 129.3 (*C−18’*), 132.9 (*C−7’*), 133.7 (*C−2’*), 136.3 (*C−4*), 144.0 (*C−1’*), 150.1 (*C−2*), 153.3 (*C−10, 12’*), 167.5 (*C−20*):

HRMS (ESI) *m/z*: 458.4807 [M+H]^+^;

Anal. Cald. for C_28_H_19_N_5_O_2_: C, 73.51; H, 4.19; N, 15.31 %. Found: C, 73.48; H, 4.21; N, 15.28 %.

**S8. Compound *5(h)*:** *2-(3-chlorophenyl)-1-(4-(phenyldiazenyl)phenyl)-1,4-dihydroimidazo[4,5-b]indole*

Compound 5*(h)* was prepared in 85% yield from 4*-*amino-functionalized azo benzene (*1*) (1 mmol), 3-chlorobenzaldehyde *2*(*h*) (1 mmol), indoline-2,3-dione (*3*) (1 mmol), ammonium acetate (*4*) (1 mmol) and (5 mol%) of L-proline in 10 ml of ethanol was irradiated through an ultrasonic wave at room temperature for the 17 minutes; Yellowish Solid; M.P.: 152-154°C;

**FTIR (ATR, υ, cm^−1^):** 3338 (N−H str.), 3087 (C−H, sp^2^), 2932 (C−H_Asym_, sp^3^), 2867 (C−H_Sym_, sp^3^), 1652 (C=C/C=N), 1579 (N=N), 1603, 1499, 1471 (CC ring str.), 702 (C−Cl);

^1^H NMR (400 MHz, CDCl_3_): δ_H_ ppm 7.28-7.43 (4H, m), 7.49-7.62 (5H, m), 7.74-7.88 (4H, m), 7.92-8.28 (4H, m), 9.95 (1H, s);

^13^C NMR (100 MHz, CDCl_3_): δ 111.3 (*C−5*), 114.4 (*C−8, 8’*), 117.0 (*C−4’*), 118.3 (*C−5’*), 122.2 (*C−13, 13’*), 124.2 (*C−9, 9’*), 125.7 (*C−16*), 127.0 (*C−19*), 127.1 (*C−17’*), 127.8 (*C−15*), 128.2 (*C−6, 14, 14’*), 128.4 (*C−6’*), 128.6 (*C−17*), 128.7 (*C−18’*), 130.4 (*C−18*), 132.9 (*C−7’*), 133.7 (*C−2’*), 136.3 (*C−4*), 144.0 (*C−1’*), 150.1 (*C−2*), 153.3 (*C−10, 12’*)

HRMS (ESI) *m/z*: 447.9305 [M+H]^+^;

Anal. Cald. for C_27_H_18_ClN_5_: C, 72.40; H, 4.05; N, 15.64 %. Found: C, 72.38; H, 4.08; N, 15.66 %.

**S9. Compound *5(i)*:** *1-(4-(phenyldiazenyl)phenyl)-2-m-tolyl-1,4-dihydroimidazo[4,5-b]indole*

Compound *5(i)* was prepared in 82% yield from 4*-*amino-functionalized azo benzene (*1*) (1 mmol), 3-methylbenzaldehyde *2*(*i*) (1 mmol), indoline-2,3-dione (*3*) (1 mmol), ammonium acetate (*4*) (1 mmol) and (5 mol%) of L-proline in 10 ml of ethanol was irradiated through an ultrasonic wave at room temperature for the 20 minutes; Yellowish Solid; M.P.: 147-149°C;

**FTIR (ATR, υ, cm^−1^):** 3328 (N−H str.), 3097 (C−H, sp^2^), 2933 (C−H_Asym_, sp^3^), 2861 (C−H_Sym_, sp^3^), 1642 (C=C/C=N), 1584 (N=N), 1617, 1494, 1456 (CC ring str.);

^1^H NMR (400 MHz, CDCl_3_): δ_H_ ppm 2.26-2.27 (3H, m), 7.27-7.43 (4H, m), 7.49-7.62 (5H, m), 7.80-7.88 (4H, m), 7.92-8.28 (4H, m), 9.91 (1H, s);

^13^C NMR (100 MHz, CDCl_3_): δ 21.3 (*C−20*), 111.3 (*C−5*), 114.4 (*C−8, 8’*), 117.0 (*C−4’*), 118.3 (*C−5’*), 122.2 (*C−13, 13’*), 124.2 (*C−9, 9’*), 125.7 (*C−16*), 127.1 (*C−17’*), 127.8 (*C−15, 17*), 128.0 (*C−18’*), 128.1 (*C−19*), 128.2 (*C−6, 14, 14’*), 128.4 (*C−6’*), 132.9 (*C−7’*), 133.7 (*C−2’*), 134.8 (*C−18*), 136.3 (*C−4*), 144.0 (*C−1’*), 150.1 (*C−2*), 153.3 (*C−10, 12’*)

HRMS (ESI) *m/z*: 428.5123 [M+H]^+^;

Anal. Cald. for C_28_H_21_N_5_: C, 78.67; H, 4.95; N, 16.38 %. Found: C, 78.72; H, 4.92; N, 16.41%.

**S10. Compound *5(j)*:** ***2-(3-nitrophenyl)-1-(4-(phenyldiazenyl)phenyl)-1,4-dihydro imidazo[4,5-b]indole***

Compound *5(j)* was prepared in 94% yield from *4-*amino-functionalized azo benzene (*1*) (1 mmol), 3-nitrobenzaldehyde *2*(*j*) (1 mmol), indoline-2,3-dione (*3*) (1 mmol), ammonium acetate (*4*) (1 mmol) and (5 mol%) of L-proline in 10 ml of ethanol was irradiated through an ultrasonic wave at room temperature for the 16 minutes; Brownish Solid; M.P.: 147-149°C;

**FTIR (ATR, υ, cm^−1^):** 3335 (N−H str.), 3089 (C−H, sp^2^), 2926 (C−H_Asym_, sp^3^), 2867 (C−H_Sym_, sp^3^), 1642 (C=C/C=N), 1585 (N=N), 1546 (N−O str.), 1609, 1493, 1465 (CC ring str.);

^1^H NMR (400 MHz, CDCl_3_): δ_H_ ppm 7.30-7.46 (4H, m), 7.52-7.67 (5H, m), 7.70-7.83 (4H, m), 7.89-8.28 (4H, m), 9.91 (1H, s);

^13^C NMR (100 MHz, CDCl_3_): δ 111.3 (*C−5*), 114.4 (*C−8, 8’*), 117.0 (*C−4’*), 117.7 (*C−19*), 118.3 (*C−5’*), 119.3 (*C−17*), 122.2 (*C−13, 13’*), 124.2 (*C−9, 9’*), 125.7 (*C−16*), 127.1 (*C−17’*), 127.8 (*C−15*), 128.2 (*C−6, 14, 14’*), 128.4 (*C−6’*), 129 (*C–18’),* 132.9 (*C−7’*), 133.7 (*C−2’*), 136.3 (*C−4*), 139.5 (*C−18*), 144.0 (*C−1’*), 150.1 (*C−2*), 153.3 (*C−10, 12’*);

HRMS (ESI) *m/z*: 459.4732 [M+H]^+^;

Anal. Cald. for C_27_H_18_N_6_O_2_: C, 70.73; H, 3.96; N, 18.33 %. Found: C, 70.69; H, 3.99; N, 18.28 %.

**S11. Compound *5(k)*: *2-(3-methoxyphenyl)-1-(4-(phenyldiazenyl)phenyl)-1,4-dihydroimidazo[4,5-b]indole***

Compound *5(k)* was prepared in 90% yield from 4*-*amino-functionalized azo benzene (*1*) (1 mmol), 3-methoxybenzaldehyde *2*(*k*) (1 mmol), indoline-2,3-dione (*3*) (1 mmol), ammonium acetate (*4*) (1 mmol) and (5 mol%) of L-proline in 10 ml of ethanol was irradiated through an ultrasonic wave at room temperature for the 14 minutes; Brownish Solid; M.P.: 154-156°C;

**FTIR (ATR, υ, cm^−1^):** 3334 (N−H str.), 3091 (C−H, sp^2^), 2948, 2933 (C−H_Asym_, sp^3^), 2871, 2858 (C−H_Sym_, sp^3^), 1639 (C=C/C=N), 1577 (N=N), 1613, 1487, 1462 (CC ring str.), 1051 (C−O);

^1^H NMR (400 MHz, CDCl_3_): δ_H_ ppm 3.81 (3H, s), 7.09-7.48 (4H, m), 7.51-7.59 (4H, m), 7.64-7.83 (4H, m), 7.89-8.28 (5H, m), 9.92 (1H, s);

^13^C NMR (100 MHz, CDCl_3_): δ 56.0 (*C−21*), 108.5 (*C−17*), 111.3 (*C−5*), 114.4 (*C−8, 8’*), 116.7 (*C−19*), 117.0 (*C−4’*), 118.3 (*C−5’*), 122.2 (*C−13, 13’*), 124.2 (*C−9, 9’*), 125.7 (*C−16*), 127.1 (*C−17’*), 127.8 (*C−15*), 128.2 (*C−6, 14, 14’*), 128.4 (*C−6’*), 129.6 (*C−18’*), 132.9 (*C−7’*), 133.7 (*C−2’*), 136.3 (*C−4*), 144.0 (*C−1’*), 150.1 (*C−2*), 153.3 (*C−10, 12’*), 158.8 (*C−18*);

HRMS (ESI) *m/z*: 444.5028 [M+H]^+^;

Anal. Cald. for C_28_H_21_N_5_O: C, 75.83; H, 4.77; N, 15.79 %. Found: C, 75.79; H, 4.75; N, 15.81 %.

**S12. Compound *5(l)*:** ***3-(1-(4-(phenyldiazenyl)phenyl)-1,4-dihydroimidazo[4,5-b]indol-2-yl)phenol***

Compound 5*(l)* was prepared in 84% yield from 4*-*amino-functionalized azo benzene (*1*) (1 mmol), 3-hydroxybenzaldehyde *2*(*l*) (1 mmol), indoline-2,3-dione (*3*) (1 mmol), ammonium acetate (*4*) (1 mmol) and (5 mol%) of L-proline in 10 ml of ethanol was irradiated through an ultrasonic wave at room temperature for the 19 minutes; Yellowish Solid; M.P.: 155-157°C;

**FTIR (ATR, υ, cm^−1^):** 3437 (O−H str.), 3336 (N−H str.), 3074 (C−H, sp^2^), 2935 (C−H_Asym_, sp^3^), 2871 (C−H_Sym_, sp^3^), 1653 (C=C/C=N), 1587 (N=N), 1614, 1496, 1471 (CC ring str.);

^1^H NMR (400 MHz, CDCl_3_): δ_H_ ppm 5.37 (1H, s), 7.11-7.46 (4H, m), 7.50-7.57 (5H, m), 7.64-7.88 (4H, m), 7.91-8.19 (4H, m), 9.94 (1H, s);

^13^C NMR (100 MHz, CDCl_3_): δ 110.6 (*C−17*), 111.3 (*C−5*), 114.4 (*C−8, 8’*), 114.5 (*C−19*), 117.0 (*C−4’*), 118.3 (*C−5’*), 122.2 (*C−13, 13’*), 124.2 (*C−9, 9’*), 125.7 (*C−16*), 127.1 (*C− 17’*), 127.8 (*C−15*), 128.2 (*C−6, 14, 14’*), 128.4 (*C−6’*), 129.6 (*C−18’*), 132.9 (*C−7’*), 133.7 (*C−2’*), 136.3 (*C−4*), 144.0 (*C−1’*), 150.1 (*C−2*), 153.3 (*C−10, 12’*), 154.4 (*C−18*);

HRMS (ESI) *m/z*: 430.4712 [M+H]^+^;

Anal. Cald. for C_27_H_19_N_5_O: C, 75.51; H, 4.46; N, 16.31 %. Found: C, 75.48; H, 4.48; N, 16.29 %.

**S13. Compound *5(m)*:** ***3-(1-(4-(phenyldiazenyl)phenyl)-1,4-dihydroimidazo[4,5-b]indol-2-yl)benzoic acid***

Compound ***5(m)*** was prepared in 93% yield from 4*-*amino-functionalized azo benzene (*1*) (1 mmol), 3-carboxybenzaldehyde *2*(*m*) (1 mmol), indoline-2,3-dione (*3*) (1 mmol), ammonium acetate (*4*) (1 mmol) and (5 mol%) of L-proline in 10 ml of ethanol was irradiated through an ultrasonic wave at room temperature for the 16 minutes; Brownish Solid; M.P.: 136-138°C;

**FTIR (ATR, υ, cm^−1^):** 3427 (O−H str.), 3334 (N−H str.), 3078 (C−H, sp^2^), 2934 (C−H_Asym_, sp^3^), 2867 (C−H_Sym_, sp^3^), 1644 (C=C/C=N), 1582 (N=N), 1609, 1493, 1458 (CC ring str.), 1053 (C−O);

^1^H NMR (400 MHz, CDCl_3_): δ_H_ ppm 5.34 (1H, s), 7.30-7.48 (4H, m), 7.52-7.63 (5H, m), 7.65-7.83 (4H, m), 7.89-8.28 (4H, m), 9.91 (1H, s);

^13^C NMR (100 MHz, CDCl_3_): δ 111.3 (*C−5*), 114.4 (*C−8, 8’*), 117.0 (*C−4’*), 118.3 (*C−5’*), 122.2 (*C−13, 13’*), 124.2 (*C−9, 9’*), 125.7 (*C−16*), 127.1 (*C−17’*), 127.4 (*C−17*), 127.8 (*C−15*), 128.0 (*C−18’),* 128.2 (*C−6, 14, 14’*), 128.4 (*C−6’*), 129.7 (*C−19*), 130.2 (*C−18*), 132.9 (*C−7’*), 133.7 (*C−2’*), 136.3 (*C−4*), 144.0 (*C−1’*), 150.1 (*C−2*), 153.3 (*C−10, 12’*), 167.1 (*C−20*);

HRMS (ESI) *m/z*: 458.4823 [M+H]^+^;

Anal. Cald. for C_28_H_19_N_5_O_2_: C, 73.51; H, 4.19; N, 15.31 %. Found: C, 73.47; H, 4.22; N, 15.48 %.

**S14. Compound *5(n)*:** *2-(4-chlorophenyl)-1-(4-(phenyldiazenyl)phenyl)-1,4-dihydroimidazo[4,5-b]indole*

Compound 5*(n)* was prepared in 91% yield from 4*-*amino-functionalized azo benzene (*1*) (1 mmol), 4-chlorobenzaldehyde *2*(*n*) (1 mmol), indoline-2,3-dione (*3*) (1 mmol), ammonium acetate (*4*) (1 mmol) and (5 mol%) of L-proline in 10 ml of ethanol was irradiated through an ultrasonic wave at room temperature for the 12 minutes; Yellowish Solid; M.P.: 142-144°C;

**FTIR (ATR, υ, cm^−1^):** 3335 (N−H str.), 3093 (C−H, sp^2^), 2927 (C−H_Asym_, sp^3^), 2872 (C−H_Sym_, sp^3^), 1646 (C=C/C=N), 1583 (N=N), 1614, 1495, 1467 (CC ring str.), 684 (C−Cl);

^1^H NMR (400 MHz, CDCl_3_): δ_H_ ppm 7.29-7.45 (4H, m), 7.53-7.69 (4H, m), 7.73-7.88 (5H, m), 7.98-8.21 (4H, m), 9.92 (1H, s);

^13^C NMR (100 MHz, CDCl_3_): δ 111.3 (*C−5*), 114.4 (*C−8, 8’*), 117.0 (*C−4’*), 118.3 (*C−5’*), 122.2 (*C−13, 13’*), 124.2 (*C−9, 9’*), 125.7 (*C−16*), 127.8 (*C−15*), 128.2 (*C−6, 14, 14’*), 128.3 (*C−17, 17’*), 128.4 (*C−6’*), 128.7 (*C−18, 18’*), 132.9 (*C−7’*), 133.7 (*C−2’,19*), 136.3 (*C−4*), 144.0 (*C−1’*), 150.1 (*C−2*), 153.3 (*C−10, 12’*);

**HRMS (ESI) *m/z*:** 448.9189 [M+H]^+^;

**Anal. Cald. for C_27_H_18_ClN_5_:** C, 72.40; H, 4.05; N, 7.92 %. **Found:** C, 72.38; H, 4.08; N, 7.89 %.

**S15. Compound *5(o)*:** *2-(4-nitrophenyl)-1-(4-(phenyldiazenyl)phenyl)-1,4-dihydroimidazo[4,5-b]indole*

Compound 5*(o)* was prepared in 87% yield from 4*-*amino-functionalized azo benzene (*1*) (1 mmol), 4-nitrobenzaldehyde *2*(*o*) (1 mmol), indoline-2,3-dione (*3*) (1 mmol), ammonium acetate (*4*) (1 mmol) and (5 mol%) of L-proline in 10 ml of ethanol was irradiated through an ultrasonic wave at room temperature for the 14 minutes; Yellowish Solid; M.P.: 152-154°C;

**FTIR (ATR, υ, cm^−1^):** 3327 (N−H str.), 3096 (C−H, sp^2^), 2934 (C−H_Asym_, sp^3^), 2874 (C−H_Sym_, sp^3^), 1636 (C=C/C=N), 1578 (N=N), 1541 (N−O str.), 1613, 1487, 1469 (CC ring str.);

^1^H NMR (400 MHz, CDCl_3_): δ_H_ ppm 7.02-7.49 (4H, m), 7.51-7.59 (4H, m), 7.72-7.83 (5H, m), 7.96-8.05 (4H, m), 9.93 (1H, s);

^13^C NMR (100 MHz, CDCl_3_): δ 111.3 (*C−5*), 114.4 (*C−8, 8’*), 117.0 (*C−4’*), 117.7 (*C− 18, 18’*), 118.3 (*C−5’*), 122.2 (*C−13, 13’*), 124.2 (*C−9, 9’*), 125.7 (*C−16*), 127.6 (*C−17, 17’*), 127.8 (*C−15*), 128.2 (*C−6, 14, 14’*), 128.4 (*C−6’*), 132.9 (*C−7’*), 133.7 (*C−2’*), 136.3 (*C−4*), 139.5 (*C−19*), 144.0 (*C−1’*), 150.1 (*C−2*), 153.3 (*C−10, 12’*);

HRMS (ESI) *m/z*: 458.4721 [M+H]^+^;

Anal. Cald. for C_27_H_18_N_6_O_2_: C, 70.73; H, 3.96; N, 18.33 %. Found: C, 70.76; H, 3.99; N, 18.35 %.

**S16. Compound *5(p)*:** *2-(4-methoxyphenyl)-1-(4-(phenyldiazenyl)phenyl)-1,4-dihydroimidazo*

*[4,5-b]indole*

Compound 5*(p)* was prepared in 88% yield from 4*-*amino-functionalized azo benzene (*1*) (1 mmol), 4-methoxybenzaldehyde *2*(*p*) (1 mmol), indoline-2,3-dione (*3*) (1 mmol), ammonium acetate (*4*) (1 mmol) and (5 mol%) of L-proline in 10 ml of ethanol was irradiated through an ultrasonic wave at room temperature for the 17 minutes; Brownish Solid; M.P.: 146-148°C;

**FTIR (ATR, υ, cm^−1^):** 3334 (N−H str.), 3088 (C−H, sp^2^), 2952, 2929 (C−H_Asym_, sp^3^), 2875, 2862 (C−H_Sym_, sp^3^), 1643 (C=C/C=N), 1581 (N=N), 1616, 1498, 1459 (CC ring str.), 1043 (C−O);

^1^H NMR (400 MHz, CDCl_3_): δ_H_ ppm 3.95 (3H, m), 7.16-7.48 (4H, m), 7.51-7.68 (4H, m), 7.74-7.88 (5H, m), 7.97-8.18 (4H, m), 9.97 (1H, s);

^13^C NMR (100 MHz, CDCl_3_): δ 56 (*C−20*), 111.3 (*C−5*), 114.3 (*C−18, 18’*), 114.4 (*C−8, 8’*), 117.0 (*C−4’*), 118.3 (*C−5’*), 122.2 (*C−13, 13’*), 124.2 (*C−9, 9’*), 125.7 (*C−16*), 127.8 (*C−15*), 128.2 (*C−6, 14, 14’*), 128.4 (*C−6’*), 128.9 (*C−17, 17’*), 132.9 (*C−7’*), 133.7 (*C−2’*), 136.3 (*C−4*), 144.0 (*C−1’*), 150.1 (*C−2*), 153.3 (*C−10, 12’*), 159.8 (*C−19*);

HRMS (ESI) *m/z*: 444.5083 [M+H]^+^;

Anal. Cald. for C_28_H_21_N_5_O: C, 75.83; H, 4.77; N, 15.79 %. Found: C, 75.79; H, 7.81; N, 15.82 %.

**S17. Compound *5(q)*:** *4-(1-(4-(phenyldiazenyl)phenyl)-1,4-dihydroimidazo[4,5-b]indol-2-yl)phenol*

Compound 5*(q)* was prepared in 86% yield from *4-*amino-functionalized azo benzene (*1*) (1 mmol), 4-hydroxybenzaldehyde *2*(*q*) (1 mmol), indoline-2,3-dione (*3*) (1 mmol), ammonium acetate (*4*) (1 mmol) and (5 mol%) of L-proline in 10 ml of ethanol was irradiated through an ultrasonic wave at room temperature for the 18 minutes; Yellowish Solid; M.P.: 145-147°C;

**FTIR (ATR, υ, cm^−1^):** 3433 (O−H str.), 3332 (N−H str.), 3067 (C−H, sp^2^), 2938 (C−H_Asym_, sp^3^), 2864 (C−H_Sym_, sp^3^), 1649 (C=C/C=N), 1592 (N=N), 1618, 1493, 1476 (CC ring str.);

^1^H NMR (400 MHz, CDCl_3_): δ_H_ ppm 5.37 (1H, s), 7.20-7.42 (4H, m), 7.53-7.69 (4H, m), 7.73-7.91 (5H, m), 7.98-8.20 (4H, m), 9.92 (1H, s);

^13^C NMR (100 MHz, CDCl_3_): δ 111.3 (*C−5*), 114.4 (*C−8, 8’*), 115.7 (*C−18, 18’*), 117.0 (*C−4’*), 118.3 (*C−5’*), 122.2 (*C−13, 13’*), 124.2 (*C−9, 9’*), 125.7 (*C−16*), 127.8 (*C−15*), 128.2 (*C−6, 14, 14’*), 128.4 (*C−6’*), 128.9 (*C−17, 17’*), 132.9 (*C−7’*), 133.7 (*C−2’*), 136.3 (*C−4*), 144.0 (*C−1’*), 150.1 (*C−2*), 153.3 (*C−10, 12’*), 157.4 (*C−19*);

HRMS (ESI) *m/z*: 430.4795 [M+H]^+^;

Anal. Cald. for C_27_H_19_N_5_O: C, 75.51; H, 4.46; N, 16.31 %. Found: C, 75.48; H, 4.45; N, 16.29 %.

S18. Compound *5(r)*: ***4-(1-(4-(phenyldiazenyl)phenyl)-1,4-dihydroimidazo[4,5-b]indol-2-yl)benzoic acid***

Compound 5*(r)* was prepared in 89% yield from 4*-*amino-functionalized azo benzene (*1*) (1 mmol), 4-carboxybenzaldehyde *2*(*r*) (1 mmol), indoline-2,3-dione (*3*) (1 mmol), ammonium acetate (*4*) (1 mmol) and (5 mol%) of L-proline in 10 ml of ethanol was irradiated through an ultrasonic wave at room temperature for the 13 minutes; Yellowish Solid; M.P.: 141-143°C;

**FTIR (ATR, υ, cm^−1^):** 3424 (O−H str.), 3331 (N−H str.), 3075 (C−H, sp^2^), 2928 (C−H_Asym_, sp^3^), 2872 (C−H_Sym_, sp^3^), 1649 (C=C/C=N), 1578 (N=N), 1613, 1498, 1463 (CC ring str.), 1047 (C−O);

^1^H NMR (400 MHz, CDCl_3_): δ_H_ ppm 5.39 (1H, s), 7.38-7.49 (4H, m), 7.56-7.71 (4H, m), 7.79-8.04 (5H, m), 8.14-8.38 (4H, m), 9.95 (1H, s);

^13^C NMR (100 MHz, CDCl_3_): δ 111.3 (*C−5*), 114.4 (*C−8, 8’*), 117.0 (*C−4’*), 118.3 (*C−5’*), 122.2 (*C−13,13’*), 124.2 (*C−9, 9’*), 125.7 (*C−16*), 127.2 (*C−19*), 127.3 (*C−17, 17’*), 127.8 (*C−15*), 128.2 (*C−6, 14, 14’*), 128.4 (*C−6’*), 129.6 (*C−18, 18’*), 132.9 (*C−7’*), 133.7 (*C−2’*), 136.3 (*C−4*), 144.0 (*C−1’*), 150.1 (*C−2*), 153.3 (*C−10, 12’*), 167.1 (*C−20*);

HRMS (ESI) *m/z*: 458.4831 [M+H]^+^;

Anal. Cald. for C_28_H_19_N_5_O_2_: C, 73.51; H, 4.19; N, 15.31 %. Found: C, 73.48; H, 4.22; N, 15.29 %.

Figure S1. The ^1^H NMR spectrum of *2-phenyl-1-(4-(phenyldiazenyl)phenyl)-1,4-dihydroimidazo[4,5-b]indole* ***5(a)***


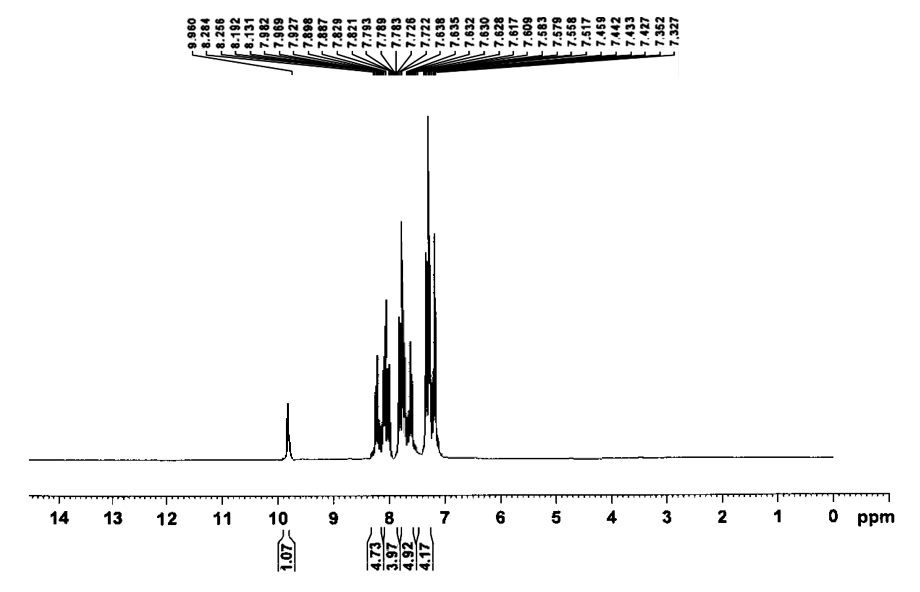


Figure S2. The ^13^C NMR spectrum of *2-phenyl-1-(4-(phenyldiazenyl)phenyl)-1,4-dihydroimidazo[4,5-b]indole* ***5(a)***


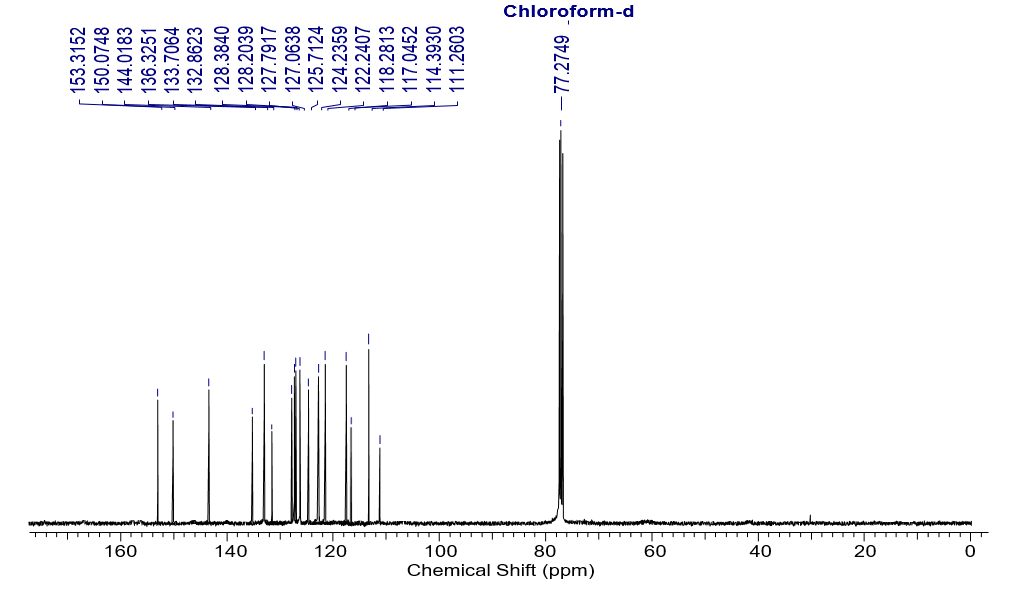


Figure S3. The ESI-MS spectrum of *2-phenyl-1-(4-(phenyldiazenyl)phenyl)-1,4-dihydroimidazo[4,5-b]indole* ***5(a)***


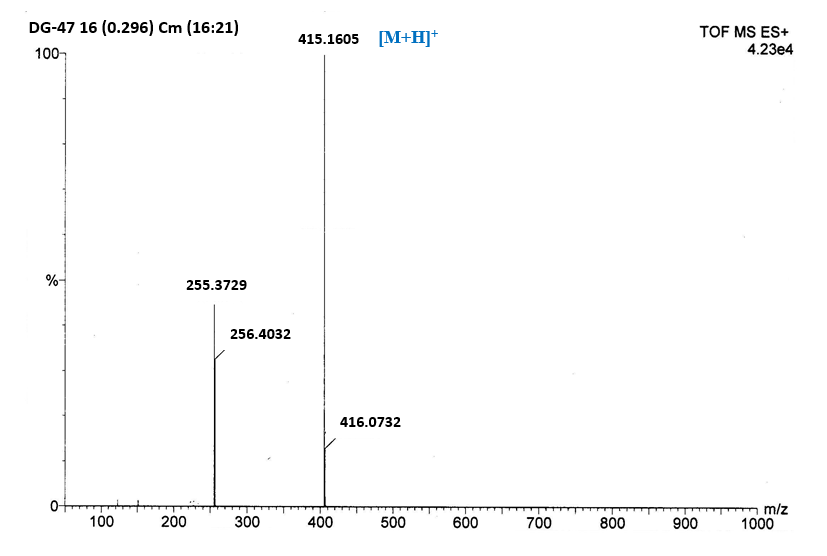


Figure S4. The ^1^H NMR spectrum of *2-(2-nitrophenyl)-1-(4-(phenyldiazenyl)phenyl)-1,4-dihydroimidazo[4,5-b]indole* ***5(d)***


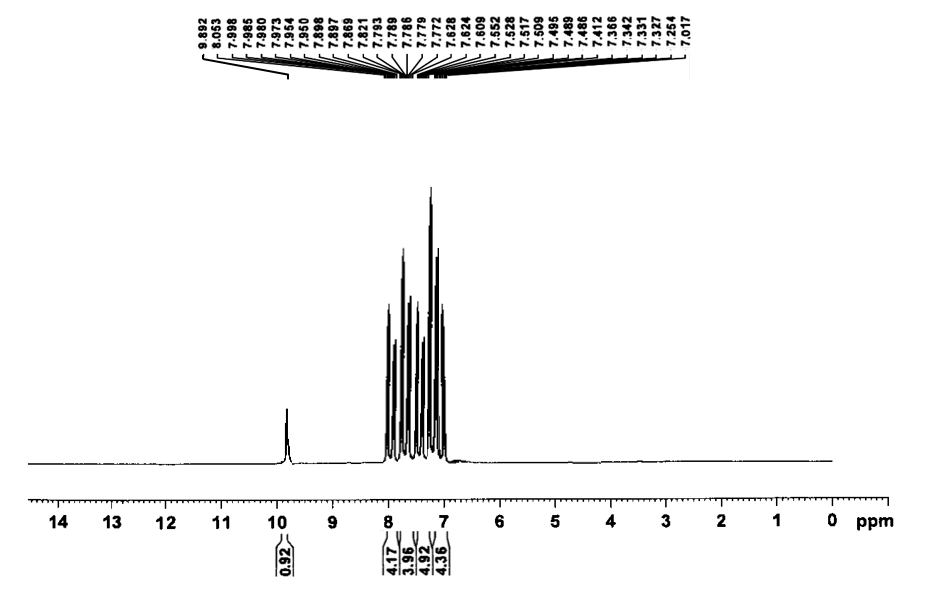


Figure S5. The ^13^C NMR spectrum of *2-(2-nitrophenyl)-1-(4-(phenyldiazenyl)phenyl)-1,4-dihydroimidazo[4,5-b]indole* ***5(d)***


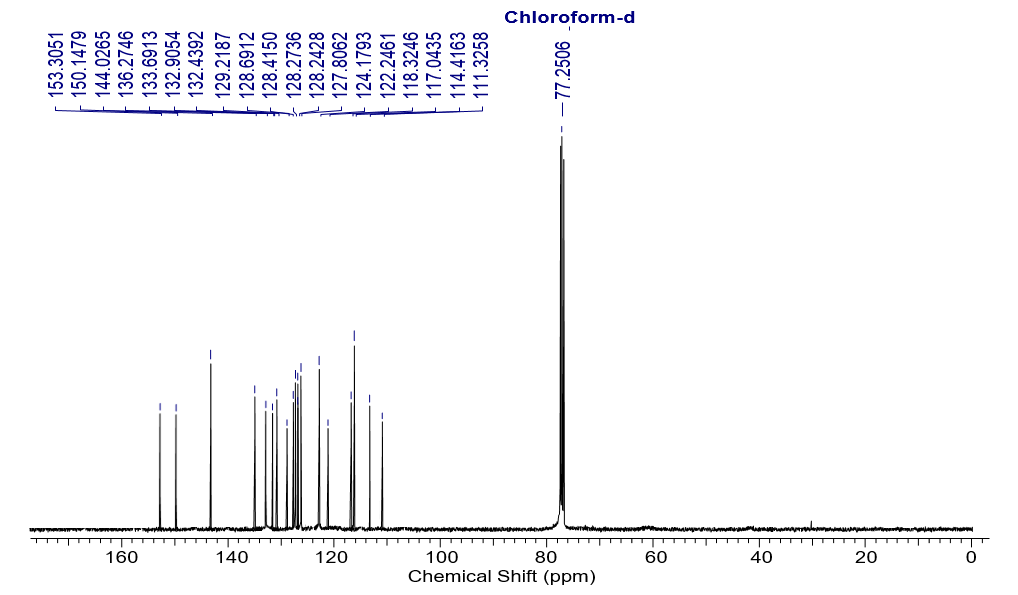


Figure S6. The ESI-MS spectrum of *2-(2-nitrophenyl)-1-(4-(phenyldiazenyl)phenyl)-1,4-dihydroimidazo[4,5-b]indole* ***5(d)***


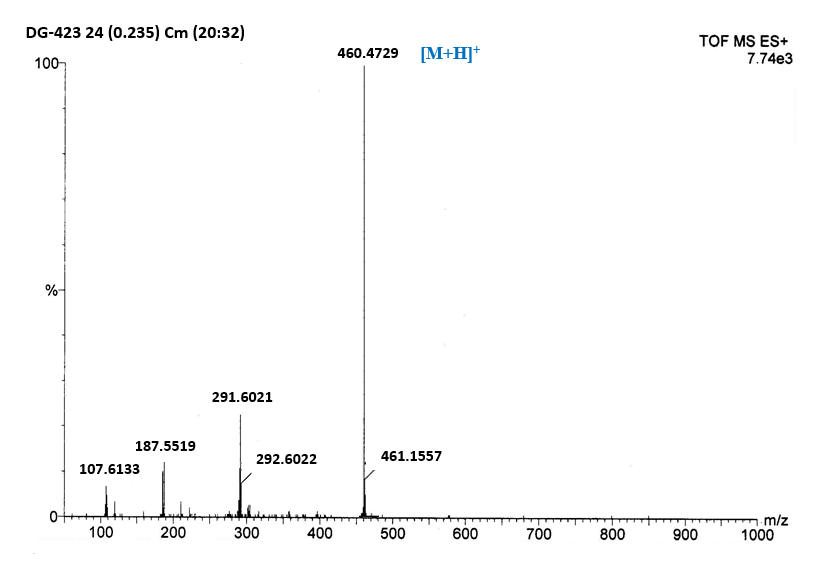


Figure S7. The ^1^H NMR spectrum of *2-(3-chlorophenyl)-1-(4-(phenyldiazenyl)phenyl)-1,4-dihydroimidazo[4,5-b]indole* ***5(h)***


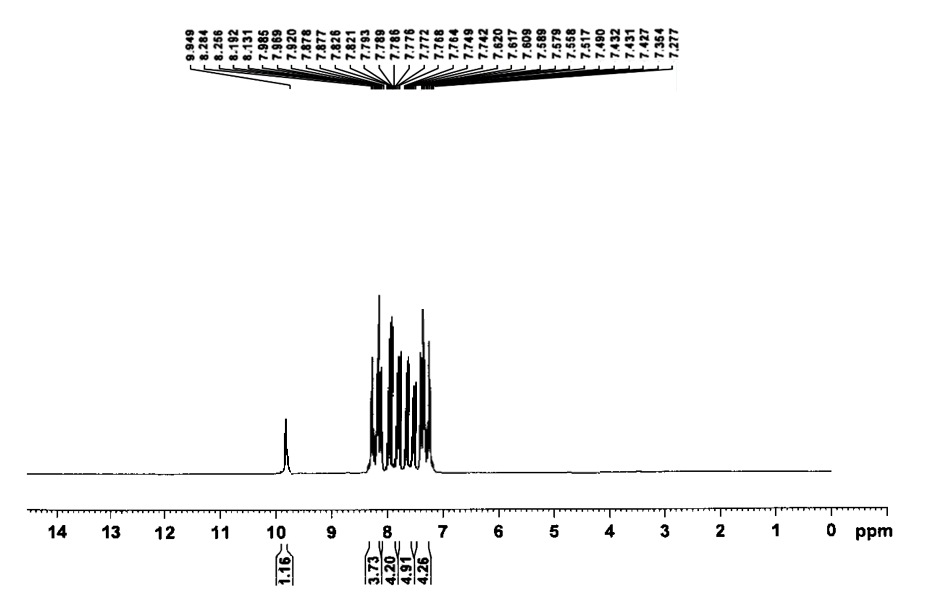


Figure S8. The ^13^C NMR spectrum of *2-(3-chlorophenyl)-1-(4-(phenyldiazenyl)phenyl)-1,4-dihydroimidazo[4,5-b]indole* ***5(h)***


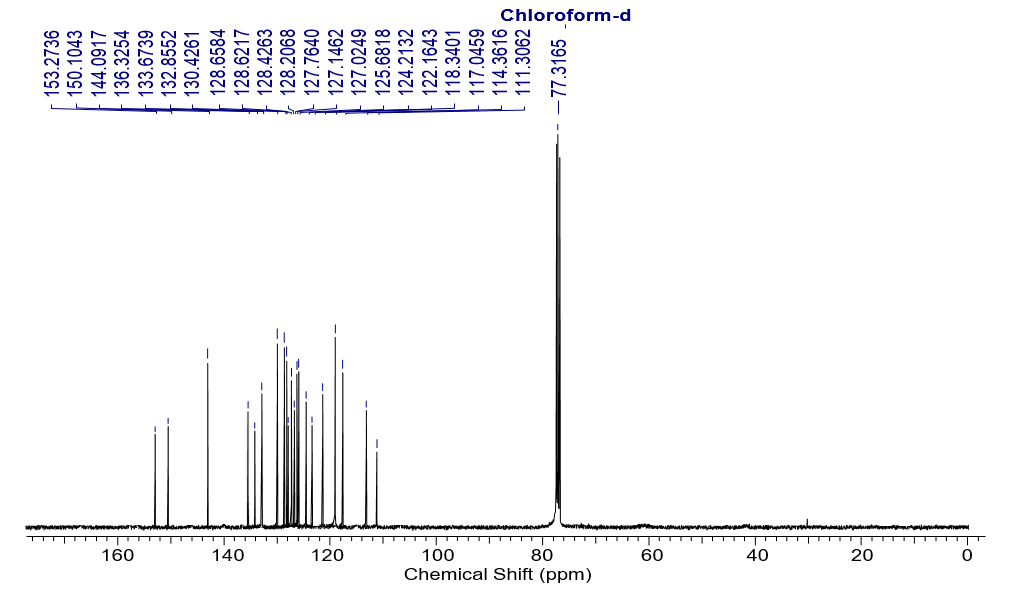


Figure S9. The ESI-MS spectrum of *2-(3-chlorophenyl)-1-(4-(phenyldiazenyl)phenyl)-1,4-dihydroimidazo[4,5-b]indole* ***5(h)***


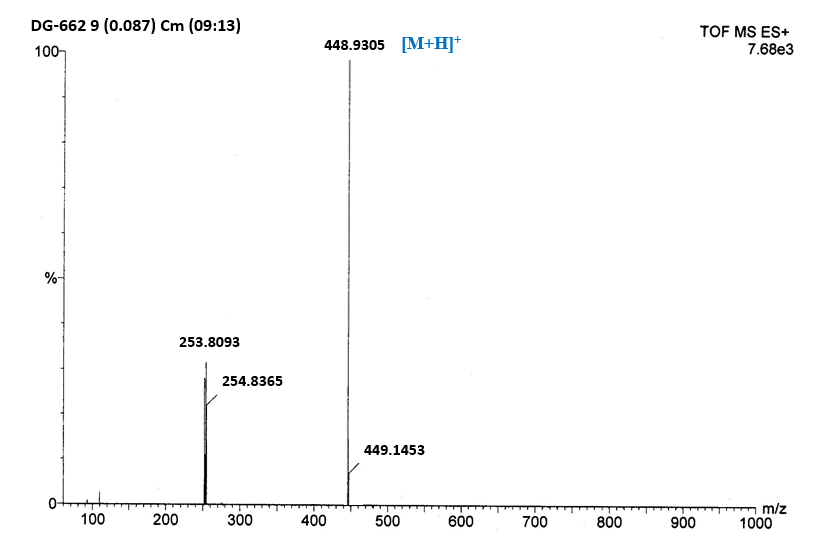


Figure S10. The ^1^H NMR spectrum of *1-(4-(phenyldiazenyl)phenyl)-2-m-tolyl-1,4-dihydroimidazo[4,5-b]indole* ***5(i)***


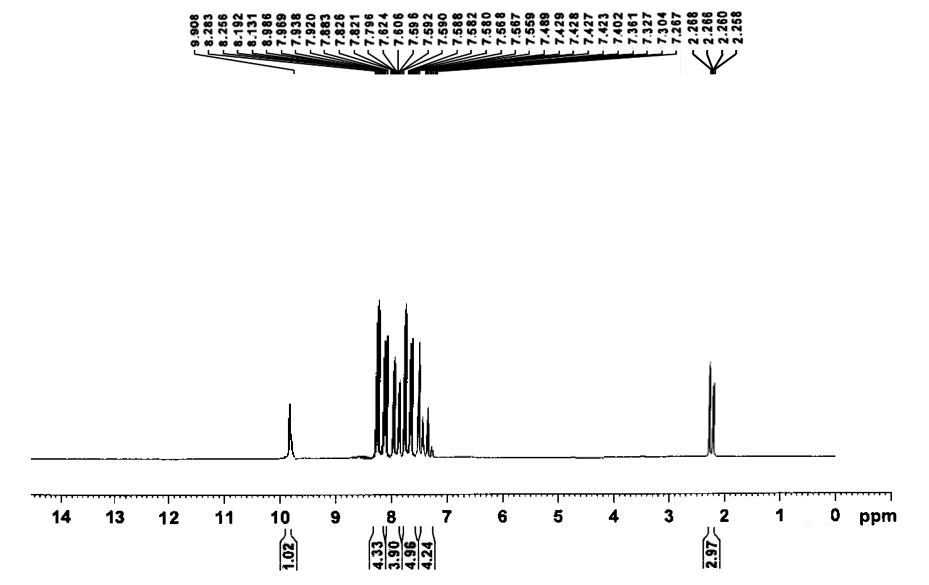


Figure S11. The ^13^C NMR spectrum of *1-(4-(phenyldiazenyl)phenyl)-2-m-tolyl-1,4-dihydroimidazo[4,5-b]indole* ***5(i)***


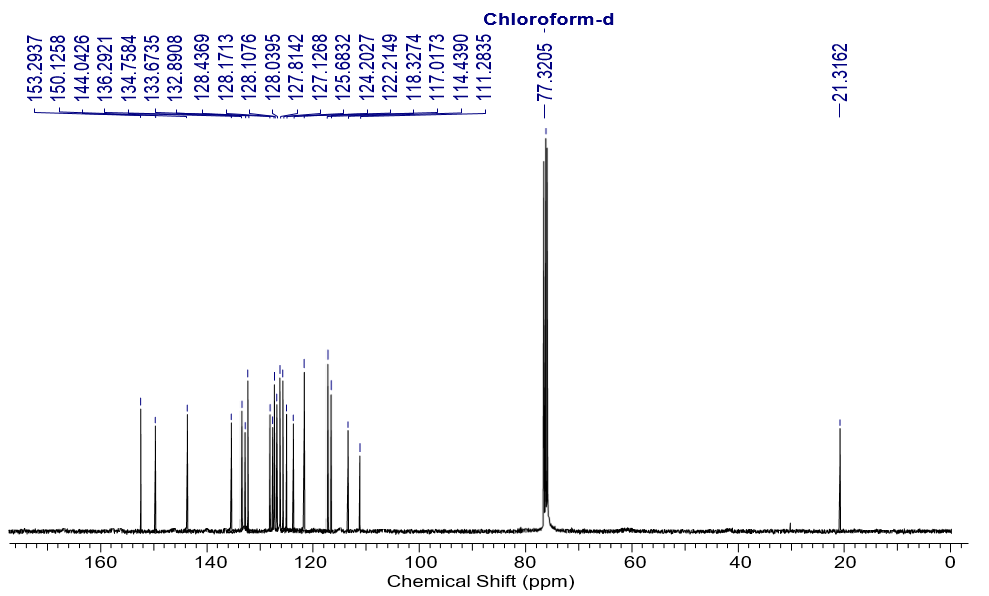


Figure S12. The ESI-MS spectrum of *1-(4-(phenyldiazenyl)phenyl)-2-m-tolyl-1,4-dihydroimidazo[4,5-b]indole* ***5(i)***


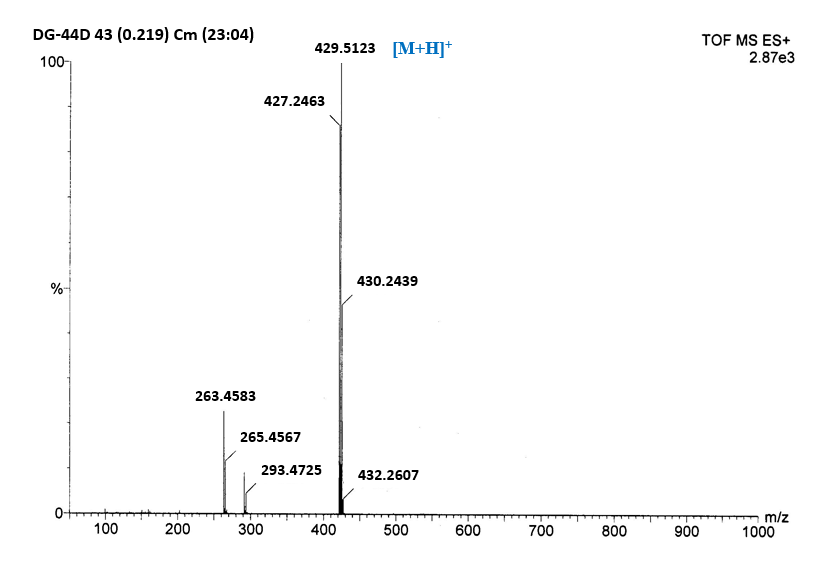


Figure S13. The ^1^H NMR spectrum of *2-(3-nitrophenyl)-1-(4-(phenyldiazenyl)phenyl)-1,4-dihydroimidazo[4,5-b]indole* ***5(j)***


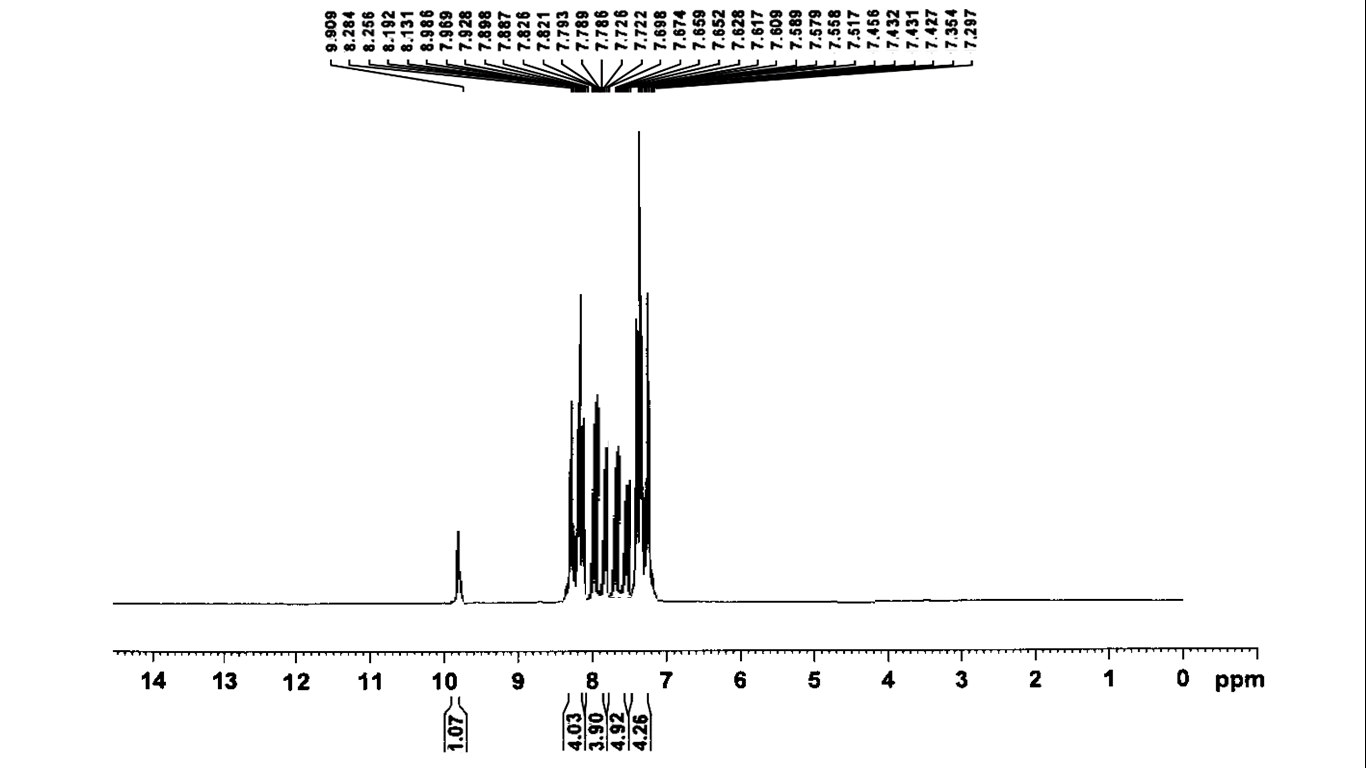


Figure S14. The ^13^C NMR spectrum of *2-(3-nitrophenyl)-1-(4-(phenyldiazenyl)phenyl)-1,4-dihydroimidazo[4,5-b]indole* ***5(j)***


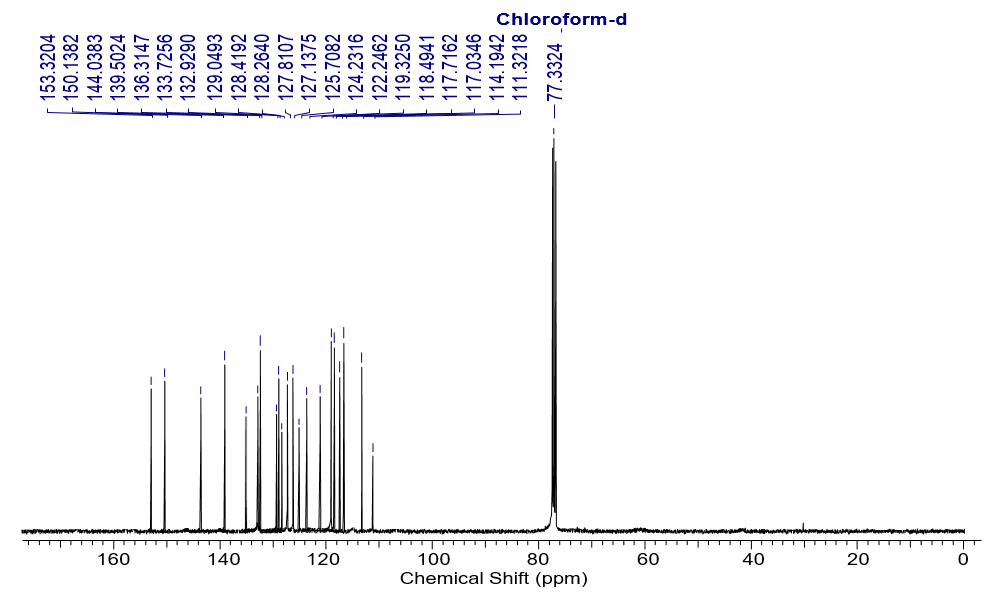


Figure S15. The ESI-MS spectrum of *2-(3-nitrophenyl)-1-(4-(phenyldiazenyl)phenyl)-1,4-dihydroimidazo[4,5-b]indole* ***5(j)***


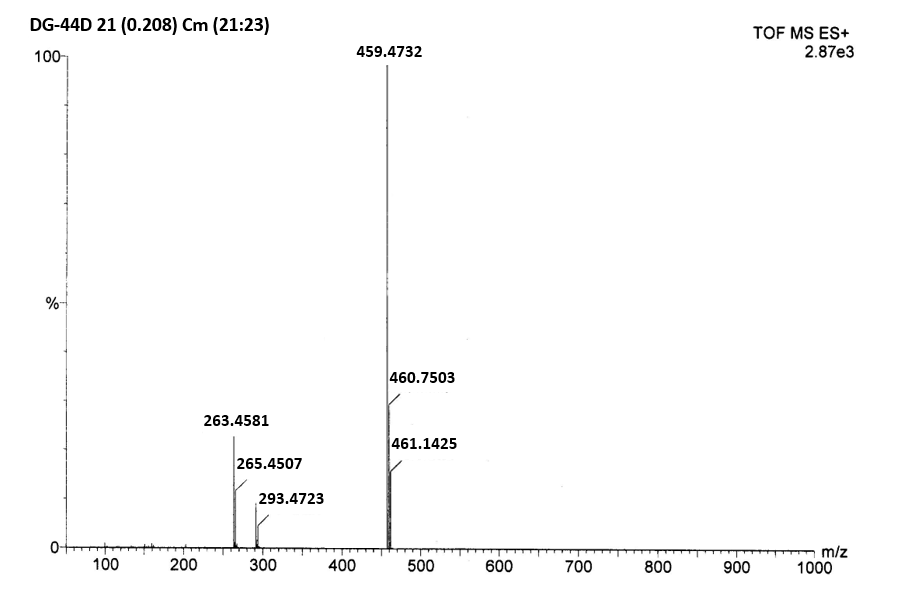


**[M+H] ^+^**

Figure S16. The ^1^H NMR spectrum of *2-(3-methoxyphenyl)-1-(4-(phenyldiazenyl)phenyl)-1,4-dihydroimidazo[4,5-b]indole* ***5(k)***


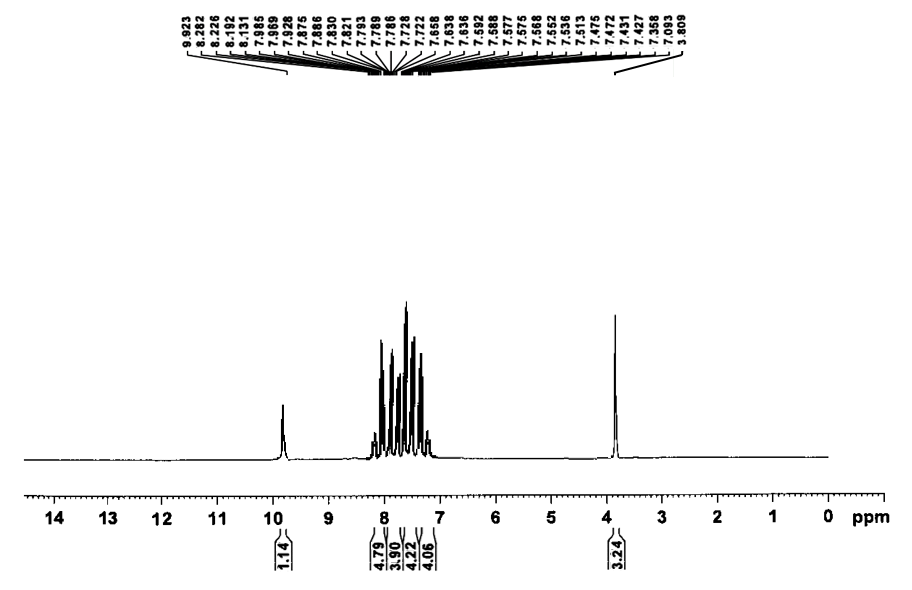


Figure S17. The ^13^C NMR spectrum of *2-(3-methoxyphenyl)-1-(4-(phenyldiazenyl)phenyl)-1,4-dihydroimidazo[4,5-b]indole* ***5(k)***


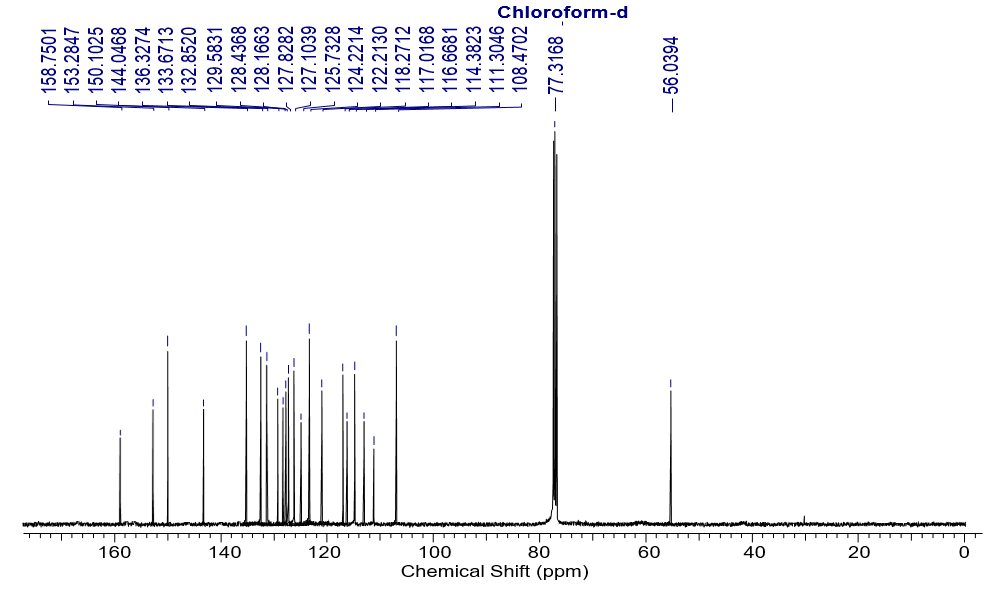


Figure S18. The ESI-MS spectrum of *2-(3-methoxyphenyl)-1-(4-(phenyldiazenyl)phenyl)-1,4-dihydroimidazo[4,5-b]indole* ***5(k)***


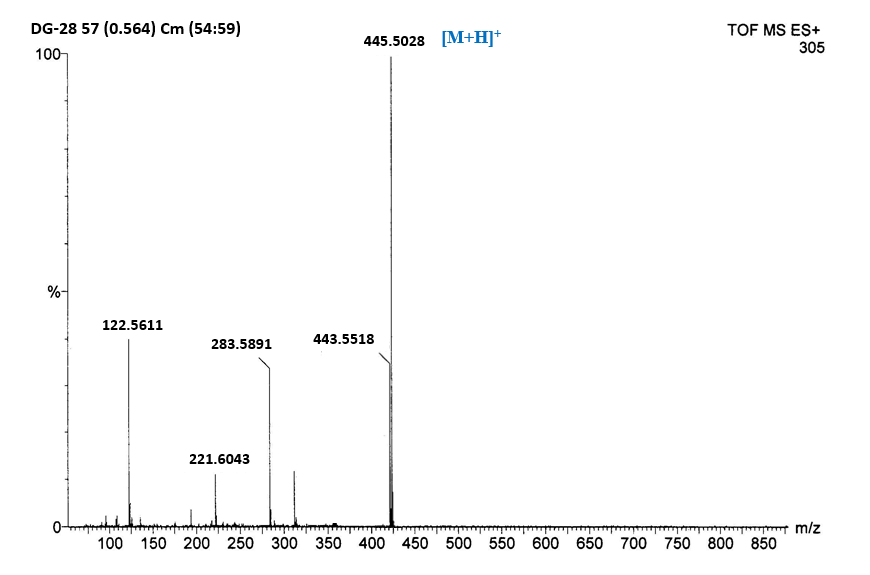


Figure S19. The ^1^H NMR spectrum of *3-(1-(4-(phenyldiazenyl)phenyl)-1,4-dihydroimidazo[4,5-b]indol-2-yl)phenol* ***5(l)***


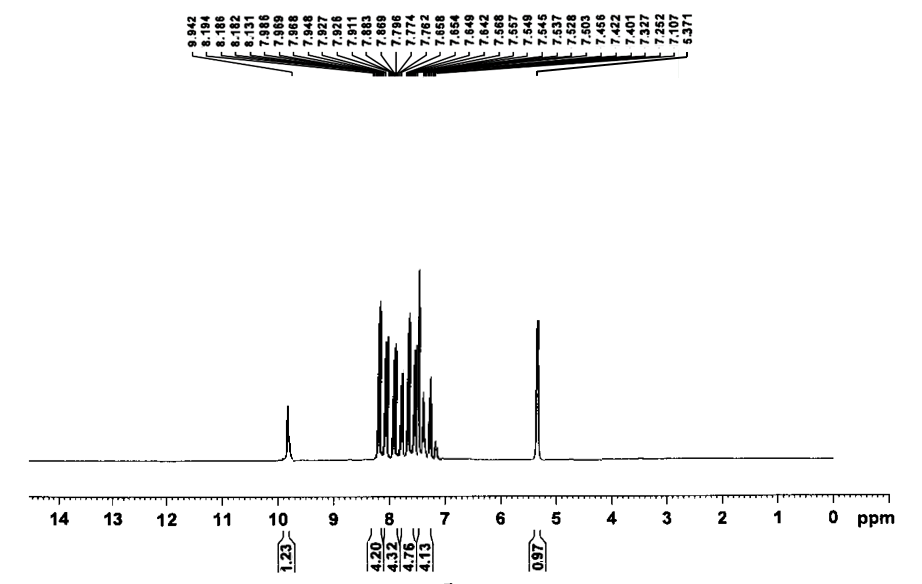


Figure S20. The ^13^C NMR spectrum of *3-(1-(4-(phenyldiazenyl)phenyl)-1,4-dihydroimidazo[4,5-b]indol-2-yl)phenol* ***5(l)***


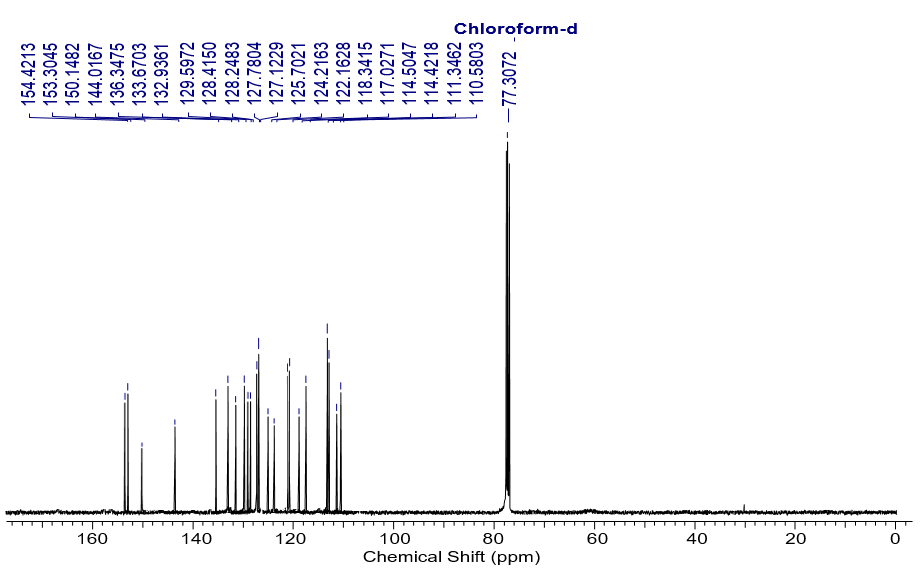


Figure S22. The ^1^H NMR spectrum of *3-(1-(4-(phenyldiazenyl)phenyl)-1,4-dihydroimidazo[4,5-b]indol-2-yl)benzoic acid* ***5(m)***


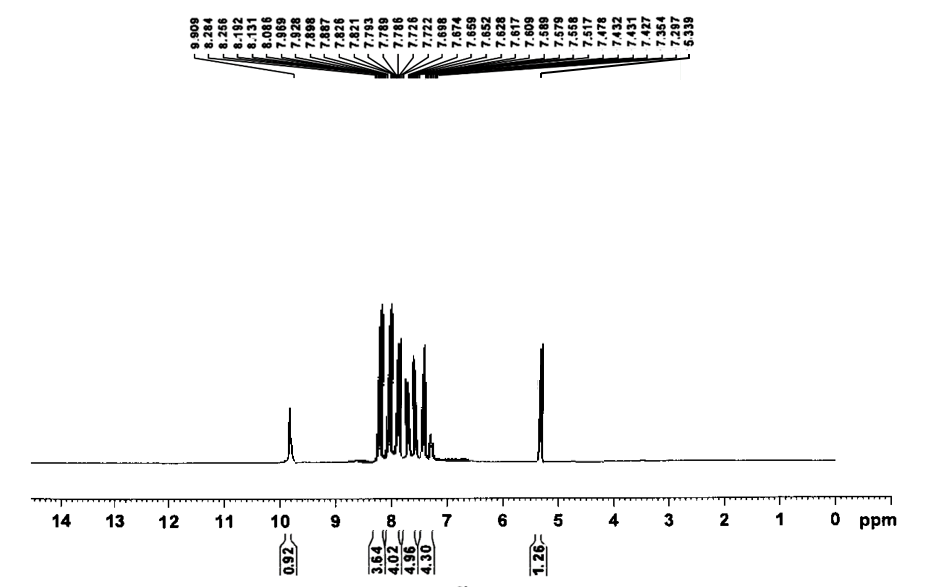


Figure S23. The ^13^C NMR spectrum of *3-(1-(4-(phenyldiazenyl)phenyl)-1,4-dihydroimidazo[4,5-b]indol-2-yl)benzoic acid* ***5(m)***


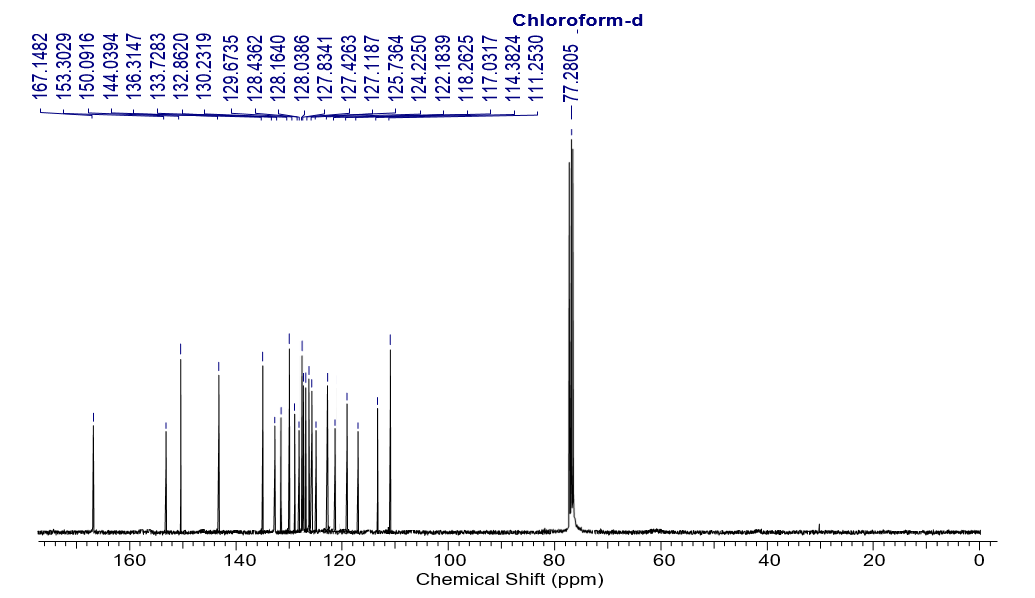


Figure S24. The ESI-MS spectrum of *3-(1-(4-(phenyldiazenyl)phenyl)-1,4-dihydroimidazo[4,5-b]indol-2-yl)benzoic acid* ***5(m)***


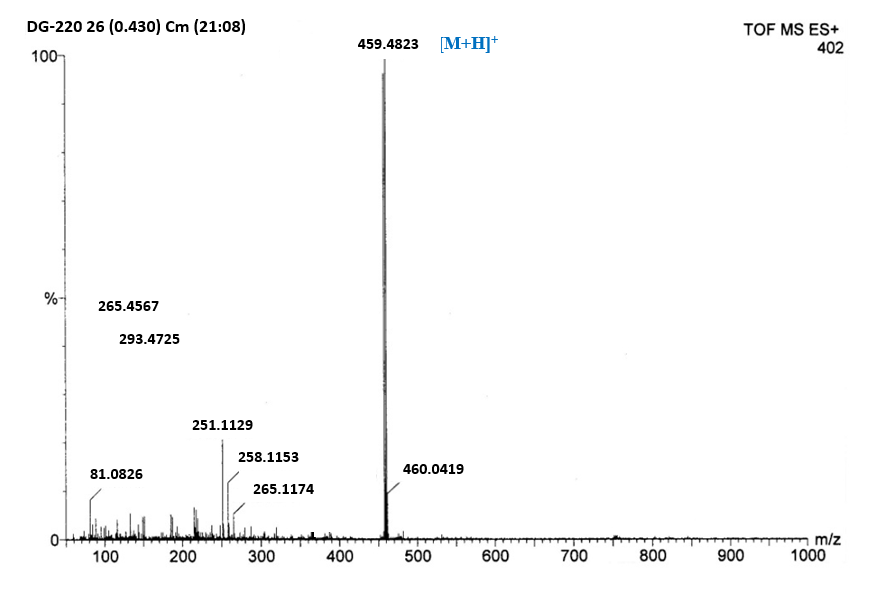


Figure S25. The ^1^H NMR spectrum of *2-(4-chlorophenyl)-1-(4-(phenyldiazenyl)phenyl)-1,4-dihydroimidazo[4,5-b]indole* ***5(n)***

*
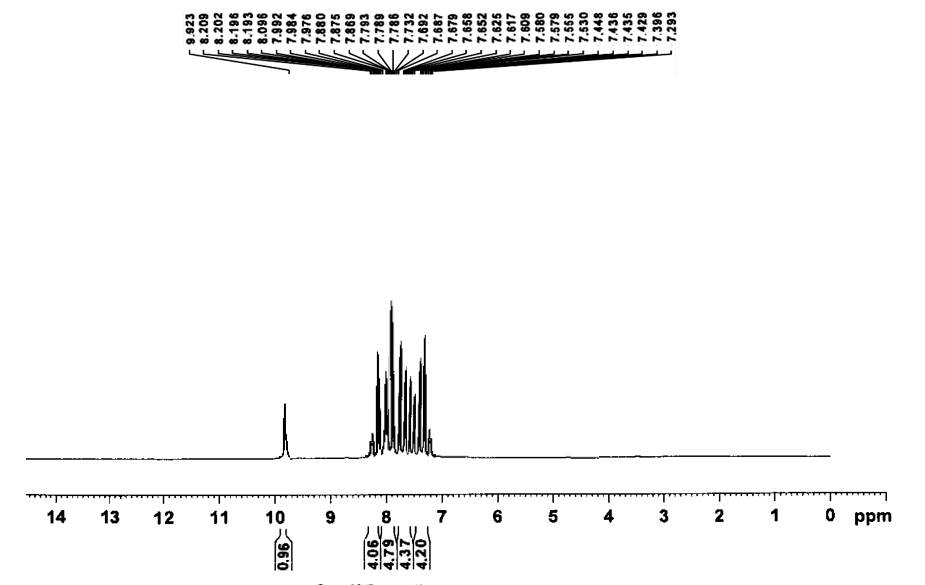
*

Figure S26. The ^13^C NMR spectrum of *2-(4-chlorophenyl)-1-(4-(phenyldiazenyl)phenyl)-1,4-dihydroimidazo[4,5-b]indole* ***5(n)***


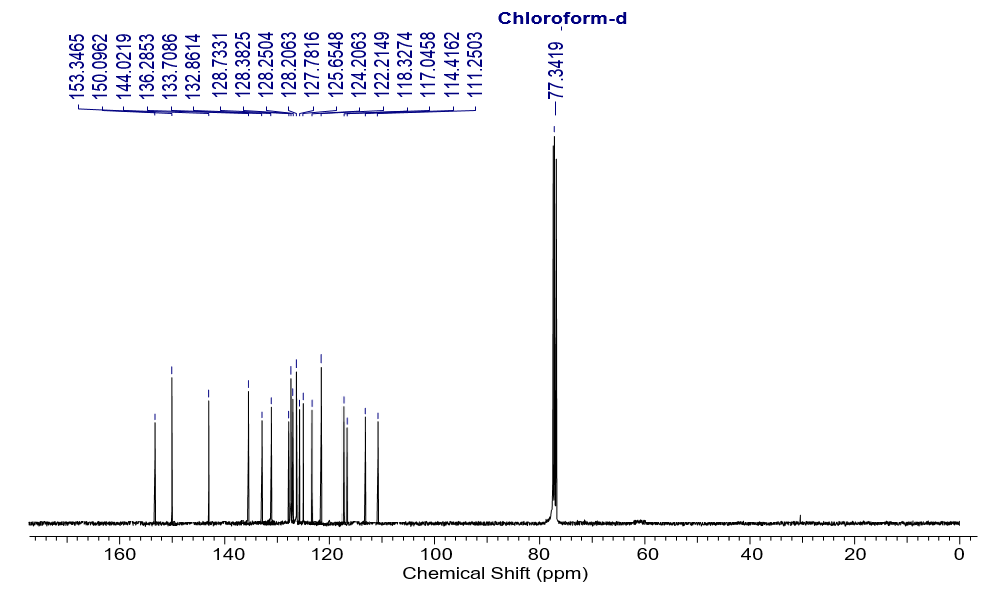


Figure S27. The ESI-MS spectrum of *2-(4-chlorophenyl)-1-(4-(phenyldiazenyl)phenyl)-1,4-dihydroimidazo[4,5-b]indole* ***5(n)***


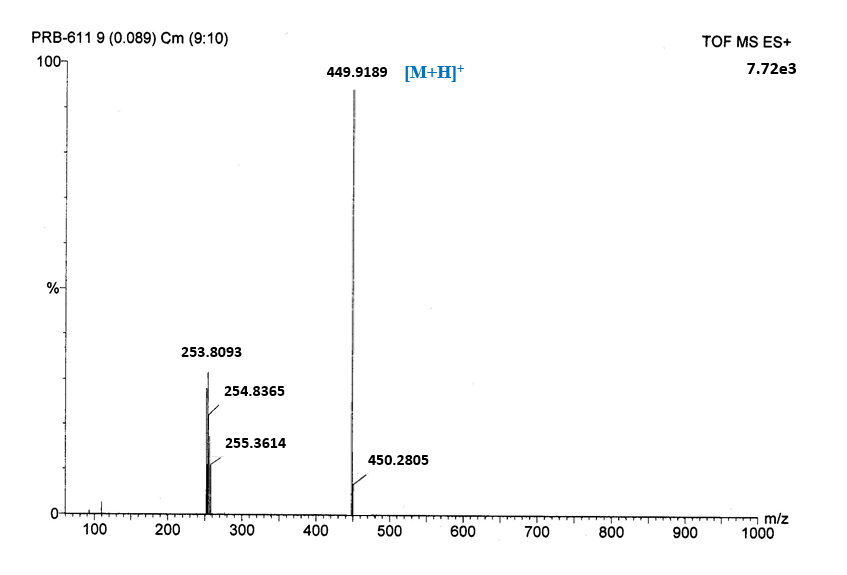


Figure S28. The ^1^H NMR spectrum of *2-(4-nitrophenyl)-1-(4-(phenyldiazenyl)phenyl)-1,4-dihydroimidazo[4,5-b]indole* ***5(o)***


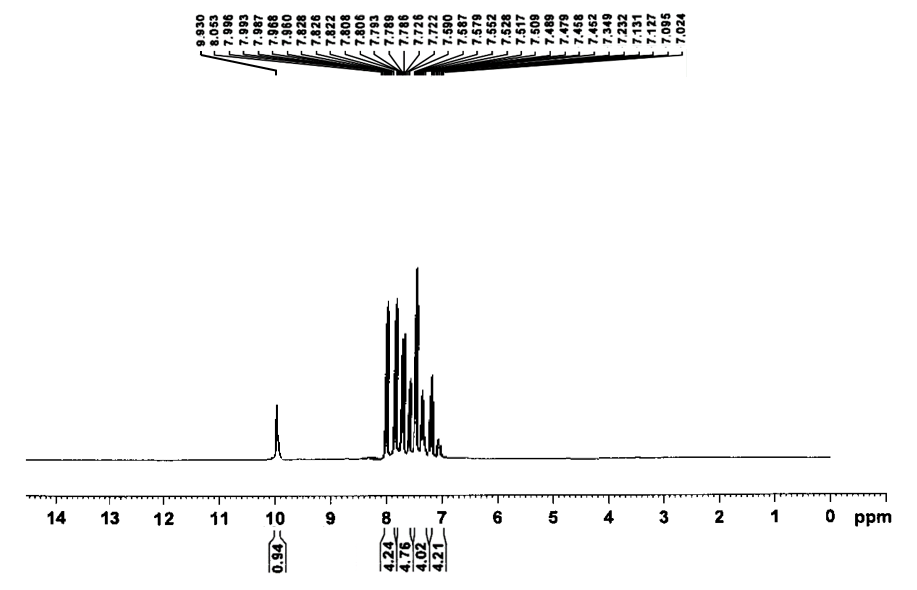


Figure S29. The ^13^C NMR spectrum of *2-(4-nitrophenyl)-1-(4-(phenyldiazenyl)phenyl)-1,4-dihydroimidazo[4,5-b]indole* ***5(o)***


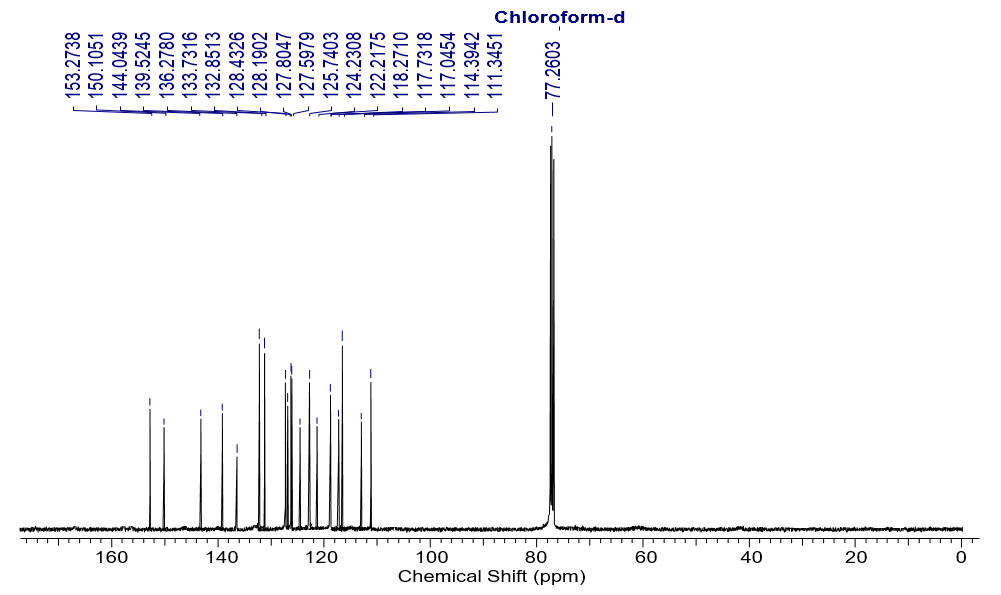


Figure S30. The ESI-MS spectrum of *2-(4-nitrophenyl)-1-(4-(phenyldiazenyl)phenyl)-1,4-dihydroimidazo[4,5-b]indole* ***5(o)***


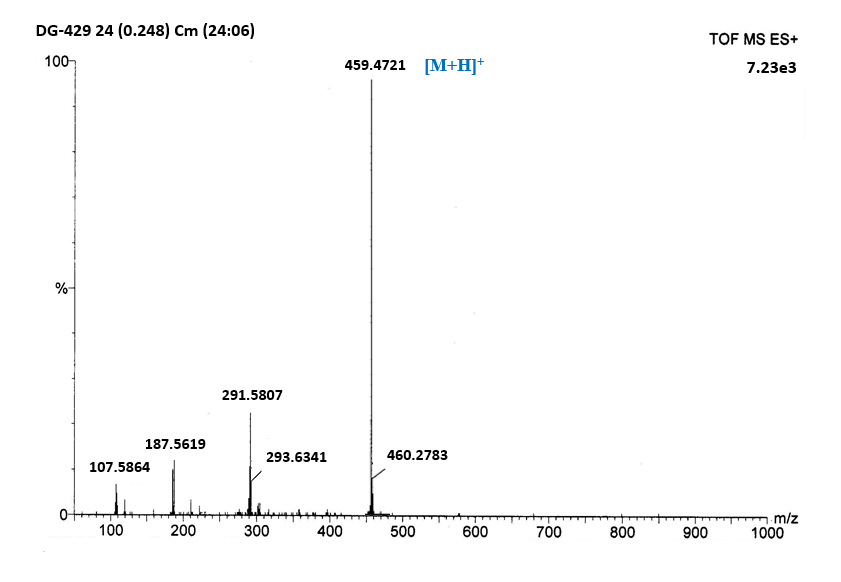


- **References:**

1. D. Geedkar, A. Kumar, G.K. Reen, P. Sharma, Titania-silica nanoparticles ensemblies assisted heterogeneous catalytic strategy for the synthesis of pharmacologically significant 2,3-diaryl-3,4-dihydroimidazo[4,5-*b*]indole scaffolds, *J Heterocyclic Chem*., **2020**, 57, 1963-1973.
2. D. Geedkar, A. Kumar, P. Sharma, Multiwalled carbon nanotubes crowned with nickel-ferrite magnetic nanoparticles assisted heterogeneous catalytic strategy for the synthesis of benzo[*d*]imidazo[2,1-*b*]thiazole scaffolds, *J Heterocyclic Chem*., **2020**, 57, 4331-4347.
3. D. Geedkar, A. Kumar, K. Kumar, P. Sharma, Hydromagnesite sheets impregnated with cobalt–ferrite magnetic nanoparticles as heterogeneous catalytic system for the synthesis of imidazo[1,2-*a*]pyridine scaffolds, *RSC Advances*, **2021**, 11, 23207-23220.
4. D. Geedkar, A. Kumar, P. Sharma, Molecular Iodine-Catalysed Synthesis of Imidazo[1,2-*a*]Pyridines: Screening of their *In Silico* Selectivity, Binding Affinity to Biological Targets and Density Functional Theory Studies Insight, *ASC Omega*, **2022**, 7, 22421-22439.
